# Plasma Promotes Fungal Cellulase Production by Regulating the Levels of Intracellular NO and Ca^2+^

**DOI:** 10.3390/ijms23126668

**Published:** 2022-06-15

**Authors:** Nan-Nan Yu, Wirinthip Ketya, Eun-Ha Choi, Gyungsoon Park

**Affiliations:** 1 Plasma Bioscience Research Center and Department of Plasma-Bio Display, Kwangwoon University, Seoul 01897, Korea; nannan19950326@163.com (N.-N.Y.); pam7794p@gmail.com (W.K.); ehchoi@kw.ac.kr (E.-H.C.); 2 Department of Electrical and Biological Physics, Kwangwoon University, Seoul 01897, Korea

**Keywords:** plasma jet, *Neurospora crassa*, cellulase, enzyme production, calcium accumulation, nitric oxide

## Abstract

For the industrial-scale production of useful enzymes by microorganisms, technological development is required for overcoming a technical bottleneck represented by poor efficiency in the induction of enzyme gene expression and secretion. In this study, we evaluated the potential of a non-thermal atmospheric pressure plasma jet to improve the production efficiency of cellulolytic enzymes in *Neurospora crassa*, a filamentous fungus. The total activity of cellulolytic enzymes and protein concentration were significantly increased (1.1~1.2 times) in media containing Avicel 24–72 h after 2 and 5 min of plasma treatment. The mRNA levels of four cellulolytic enzymes in fungal hyphae grown in media with Avicel were significantly increased (1.3~17 times) 2–4 h after a 5 min of plasma treatment. The levels of intracellular NO and Ca^2+^ were increased in plasma-treated fungal hyphae grown in Avicel media after 48 h, and the removal of intracellular NO decreased the activity of cellulolytic enzymes in media and the level of vesicles in fungal hyphae. Our data suggest that plasma treatment can promote the transcription and secretion of cellulolytic enzymes into the culture media in the presence of Avicel (induction condition) by enhancing the intracellular level of NO and Ca^2+^.

## 1. Introduction

Cellulose is a great and widespread type of renewable resource in nature, found in straw and textile waste [1]. The degraded products can be used to produce biofuels or energy for other organisms, which is of great significance to industry and nutrient cycling [2]. However, the technical level and production costs limit the use of cellulose. In addition, the waste cellulose is not only associated with environmental pollution, but also resource waste. Thus, efficient utilization of cellulose is necessary. Cellulose is a natural polymer composed of D-glucose units connected by β-1,4-glycosidic bonds [3]. Complete degradation through conventional chemical and physical methods is difficult. Currently, biodegradation using cellulases has received great attention due to its effectiveness. However, the production process of cellulases is relatively expensive and contributes to 50% of the total hydrolysis cost [4]. Therefore, finding a feasible method to increase the yield of cellulases and reduce its production costs is necessary. Cellulase belongs to an enzyme family with at least three types of enzymes: endoglucanase (endo-1, 4-β-D-glucanase, EG, EC 3.2.1.4); cellobiohydrolase or exoglucanase (exo-1, 4-β-D-glucanase, CBH, EC 3.2.1.91), and β-glucosidase (1, 4-β-D-glucosidase, BG, EC 3.2.1.21) [5]. First, endoglucanase can directly attack the amorphous region of cellulose, and then cellobiohydrolase hydrolyzes cellulose into cellobiose. Finally, cellobiose is decomposed into glucose by β-glucosidase, while β-glucosidase can also degrade some cello-oligosaccharides into cellobiose and glucose [6]. Recently, cellulase became the third most important industrial enzyme in the global enzyme market, according to statistics from 2020, and this is expected to be the case at least until 2027 [4].

Fungi are excellent candidate organisms for the bioproduction of cellulolytic enzymes (cellulases) because they produce and secrete these enzymes extracellularly for decomposing cellulosic materials (energy sources) in nature. Extracellularly secreted enzymes are more convenient for subsequent enzyme separation and extraction than intracellularly produced enzymes. In addition, the cellulase produced by fungi has a more reasonable structure, and the decomposition efficiency of the enzyme is higher [7]. 

Improvement in the production yield of fungal enzymes has been actively investigated. The production yield of fungal enzymes can be determined by examining the level of intracellular enzyme expression and the efficiency of the secretory process. Even though enzymes are highly expressed within the cell, the efficiency of extracellular secretion often causes a technical bottleneck in achieving high yields. Generation of mutant strains with high secretion capacities have been produced; however, this mutagenesis is random and uncontrollable, as well as financially and temporally expensive [8]. Recently, genetically engineered strains for the mass production of enzymes have been generated and evaluated [9]. Although genetic-based approaches have provided a reliable production system, ecological disadvantages of genetically modified organisms can represent a putative problem. Studies have also shown that numerous chemicals can promote the secretion of cellulases in fungi [10]; however, the chemicals will remain in the cultured supernatant and can be difficult to remove in subsequent enzyme separation and purification processes, which not only makes the operation more complicated, but also increases production costs.

Recently, non-thermal atmospheric pressure plasma has been applied to improve the yield of fungal secreted enzymes [11]. Plasma is considered as the 4th state of matter [12]. When the applied voltage reaches the breakdown voltage, gas molecules are ionized, resulting in a mixture of electrons, ions, atoms, and atomic groups [12,13]. Previously, we showed that nitrogen plasma can promote the secretion of α-amylase and spore germination in *Aspergillus oryzae* [14,15]. This suggests that plasma may be able to become a new means to improve enzyme production. From a long-term perspective, non-thermal atmospheric pressure plasma has excellent application prospects in enzyme bioproduction because the plasma device is simple to operate, and the cost of gas (e.g., air) used for plasma generation is low [11]. However, several problems remain to be solved to make non-thermal atmospheric pressure plasma a more reliable technique for the mass production of enzymes; for instance, addressing whether the promotion of fungal enzyme secretion by plasma is universal. Suitable treatment conditions and detailed regulatory mechanisms are not fully understood for most fungi. Therefore, we investigated the effects of plasma on cellulose production in the filamentous model fungus *Neurospora crassa* and its underlying mechanism(s) in this study. Our study not only provides new technical means for improving cellulase production, but also a better understanding of the basic theory necessary for future industrial applications of plasma. 

## 2. Results

### 2.1. Physical and Chemical Properties of Vogel’s Minimal (VM) Media Treated with the Plasma Jet

**Figure 1:**
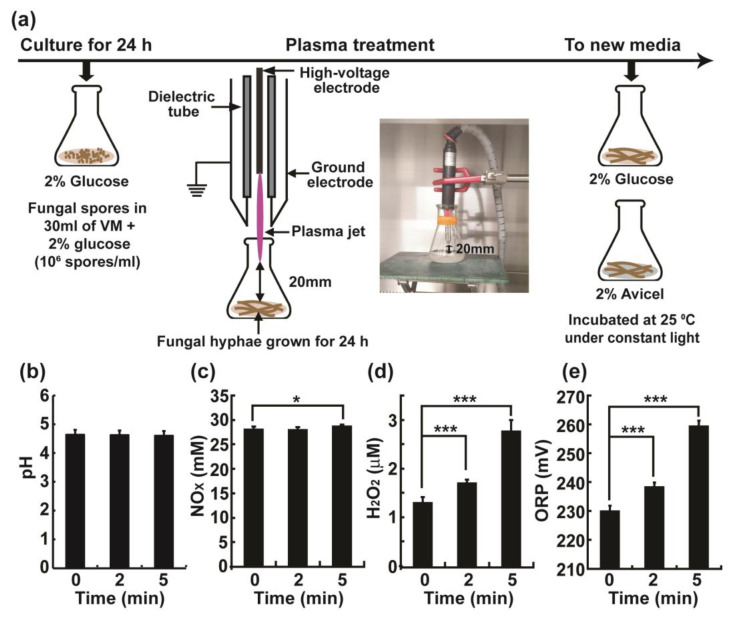
Plasma jet treatment: (**a**) Experimental scheme for treatment of 24 h-cultured *Neurospora crassa* hyphae in Vogel’s Minimal (VM) media + 2% glucose with a plasma jet. Treated fungal hyphae were transferred into non-induction (VM + 2% glucose) or induction (VM + 2% Avicel) media and further analyzed. The (**b**) pH, (**c**) NOx level, (**d**) H_2_O_2_ level, and (**e**) oxidation reduction potential (ORP) of VM media + 2% glucose after plasma jet treatment. All data points are obtained immediately after plasma jet treatment. Each value represents the mean of 9–12 replicate measurements: * *p* < 0.05, *** *p* < 0.001.

Fungal hyphae grown for 24 h were exposed to a plasma jet (Figure 1a). Immediately after the plasma treatment, the fungal hyphae were transferred to an environment for induction or no induction of cellulase expression (Figure 1a). Media containing Avicel, which is cellulose microcrystalline with a mean particle size of approximately 50 microns, typically added to liquid media to induce the expression of cellulases [16], or media containing glucose (no induction) were used. Fungal hyphae were treated with the plasma jet in VM (+2% glucose) media, indicating that the VM media were also exposed to plasma. However, the influence of the plasma-exposed medium may be subtle due to the immediate transfer of the fungal hyphae to new media (Figure 1a). The pH of the media was not significantly changed after plasma treatment and ranged between pH 4.5–4.7 (Figure 1b). This may be due to the buffering capacity of VM medium. However, the nitric oxide species (NO_X_) and hydrogen peroxide (H_2_O_2_) levels were significantly increased in the media. The concentration of NO_X_ increased by approximately 0.59 mM (28.08 mM to 28.67 mM) after 5 min of plasma treatment (Figure 1c). The concentration of H_2_O_2_ increased by 0.40 μM and 1.46 μM (1.29 μM to 1.69 μM and 2.75 μM) after 2 and 5 min of plasma treatment, respectively (Figure 1d). Finally, the oxidation-reduction potential (OPR) value increased in a treatment time-dependent manner, with an increase of 8.33 mV at 2 min (229.89 mV to 238.22 mV) and an increase of 29.33 mV at 5 min (229.89 mV to 259.22 mV) (Figure 1e). The above results showed that, although the pH of the media did not change, the NO_X_, as well as the H_2_O_2_ levels, and the redox potential were significantly increased.

### 2.2. Plasma Jet Treatment can Enhance the Production of Cellulases in N. crassa

**Figure 2:**
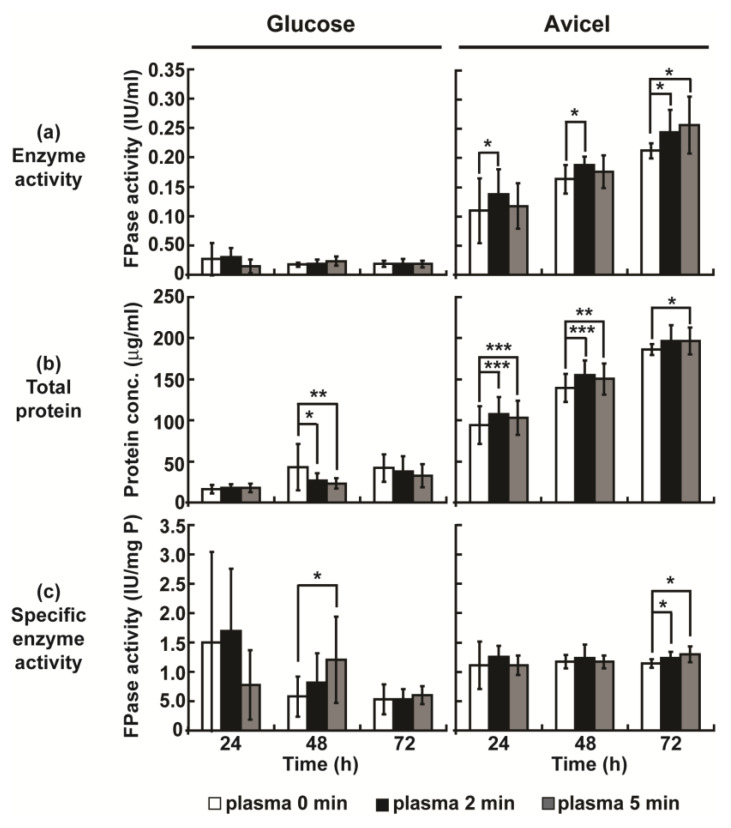
Activities of cellulolytic enzymes measured in media. Media were harvested at 24, 48, and 72 h after plasma treatment. (**a**) Filter paper enzyme (FPase) activity (total activity of cellulolytic enzymes) in media. (**b**) Total protein concentration in media. (**c**) Specific enzyme activity. Each value represents the mean of 9–12 replicate measurements: * *p* < 0.05, ** *p* < 0.01, *** *p* < 0.001.

The filter paper enzyme activity (total activity of cellulolytic enzymes) and total protein concentration in the culture media were detected at 24, 48, and 72 h after plasma treatment. Without plasma treatment, the total activity of cellulolytic enzymes degrading the filter paper was observed to be higher in Avicel media than that observed in glucose media, and the activity of the cellulolytic enzymes increased continuously with incubation time (Figure 2). Thus, the induction effect of Avicel on the production of cellulolytic enzymes was verified in our study. Our results show that plasma appears to enhance cellulolytic enzyme production mainly in media containing Avicel. In Avicel media, the total activity of cellulolytic enzymes was significantly increased after plasma treatment for 2 or 5 min, compared to that observed under no plasma treatment, for all incubation times (Figure 2a). The total protein concentration in the Avicel media condition increased significantly after plasma treatment (2 and 5 min) for all incubation times (Figure 2b). In Avicel media, specific enzyme activity (total enzyme activity divided by total protein) was increased only at 72 h (Figure 2c). However, no obvious change in total activity of cellulolytic enzymes and total protein concentration was observed in the glucose media condition (Figure 2). Our results showed an approximately 1.1–1.2-fold increase in total cellulolytic enzyme activity and total protein concentration following plasma treatment, compared to the results observed for the untreated group (Figure S1). Thus, it can be concluded that a plasma jet can promote the Avicel-induction of cellulolytic enzyme production.

To elevate the enhancement effect, we increased the treatment duration (10 min) and number of treatments (2 times). The increase in treatment duration to 10 min significantly reduced enzyme activity and protein concentration (Figure S2). Fungal hyphae were treated with plasma again 24 h after the first treatment. We observed that a second plasma treatment (2 min) significantly increased the activity of total cellulolytic enzymes after 24 h and the specific enzyme activity in all conditions (Figure S3). However, total protein concentration was reduced after plasma treatment (Figure S3). This indicates that there might be a plasma dose threshold resulting in the activation or enhancement of the enzyme production process.

**Figure 3:**
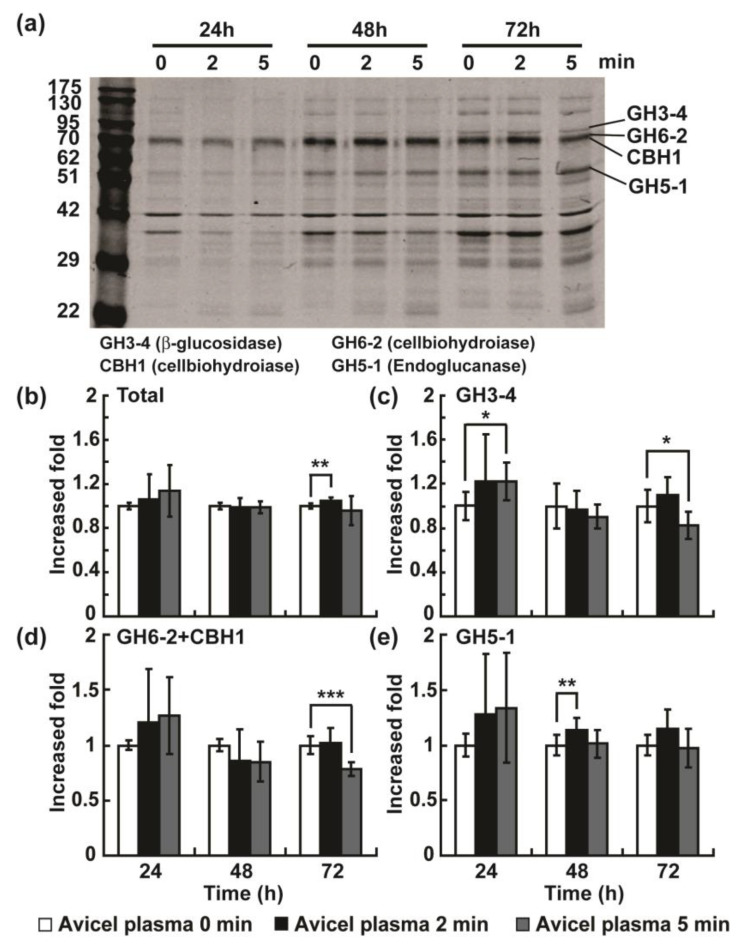
Analysis of cellulolytic enzymes in Avicel media by gel electrophoresis. The media was harvested at 24, 48, and 72 h after plasma treatment (0, 2, and 5 min). (**a**) Secreted proteins into media resolved on an SDS-PAGE gel. (**b**–**e**) The relative intensity of all 4 enzyme bands (**b**) and each protein band, namely (**c**) GH3-4, (**d**) GH6-2 + CBH1, and (**e**) GH5-1. ImageJ software (National Institute of Health, Bethesda, MD, USA) was used for quantifying the relative band intensities from the plasma-treated group compared to those from the untreated group. Each value represents the mean of six replicate measurements: * *p* < 0.05, ** *p* < 0.01, *** *p* < 0.001.

We also analyzed the protein profiles in media after plasma treatment using gel electrophoresis. The previous proteomic analysis in *N. crassa* demonstrated that the top four cellulases showing significantly increased secretion levels due to Avicel induction were two cellobiohydrolases (CBH1 and GH6-2), an endoglucanase (GH5-1), and a beta-glucosidase (GH3-4) [17,18,19]. Due to the lack of available antibodies for these enzymes, protein bands resolved on the SDS-PAGE gel were analyzed after staining. We analyzed the proteins in Avicel media because the protein level in glucose media was prohibitively low. Generally, the band intensity of overall proteins increased as a function of the induction time, indicating that secretion is induced (Figure 3a). The total intensities of the four enzyme bands estimated using ImageJ software (National Institute of Health, Bethesda, MD, USA) were slightly increased after 72 h and following a 2 min plasma treatment (Figure 3b). The intensity levels of the GH3-4 and GH5-1 bands increased at 24 and 48 h after plasma treatment for 5 min and 2 min, respectively (Figure 3c,e). Interestingly, the level of the CBH1 and GH6-2 band intensities was decreased at 72 h after plasma treatment (Figure 3d). This result indicates that the production (expression and secretion) of different cellulases may be spatiotemporally regulated because the different types of cellulolytic enzymes are involved in different sequences of action when degrading cellulose.

**Figure 4:**
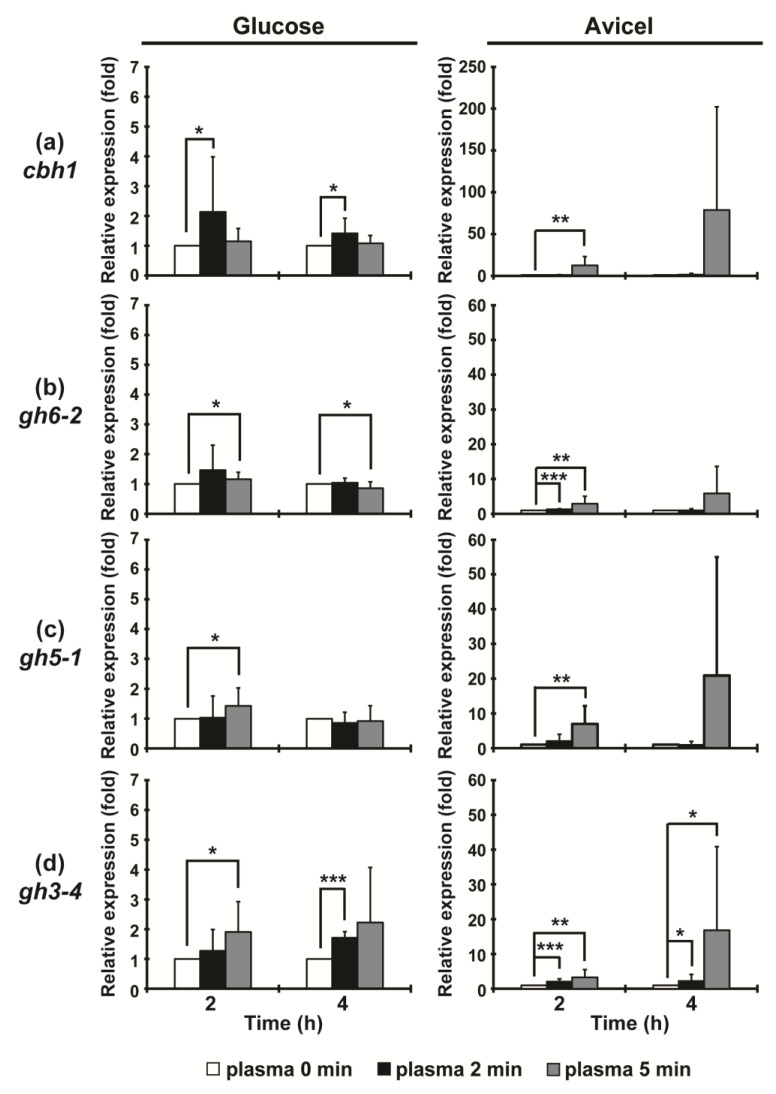
Intracellular mRNA expression of cellulolytic enzymes in fungal hyphae incubated in glucose and Avicel media at 2 and 4 h after plasma treatment (0, 2, and 5 min): (**a**) *cbh1*; (**b**) *gh6-2*; (**c**) *gh5-1*; (**d**) *gh3-4.* Each value represents the mean of 9–12 replicate measurements: * *p* < 0.05, ** *p* < 0.01, *** *p* < 0.001.

Further, we measured the mRNA expression levels of the four cellulolytic enzyme genes to assess whether plasma treatments affect the intracellular enzyme gene expression as well. In Avicel media (induction condition), the mRNA levels of the four cellulolytic enzyme genes were significantly increased (about 1.3~17-fold) in a 5 min plasma treated fungal hyphae, mostly after 24 h (Figure 4). Particularly, *gh6- 2* and *gh3-4* mRNA levels were already increased after 2 min of plasma treatment (about 1.2~2-fold) (Figure 4b,d). Interestingly, the mRNA levels of four enzymes were also slightly (significantly) elevated in several plasma treatments (about 1.2–2.2-fold) in the glucose, i.e., the non-inducing (not inducing expression of cellulolytic enzymes) medium (Figure 4).

### 2.3. Plasma Treatment Promotes Slight Vesicle Accumulation in Fungal Hyphae

**Figure 5:**
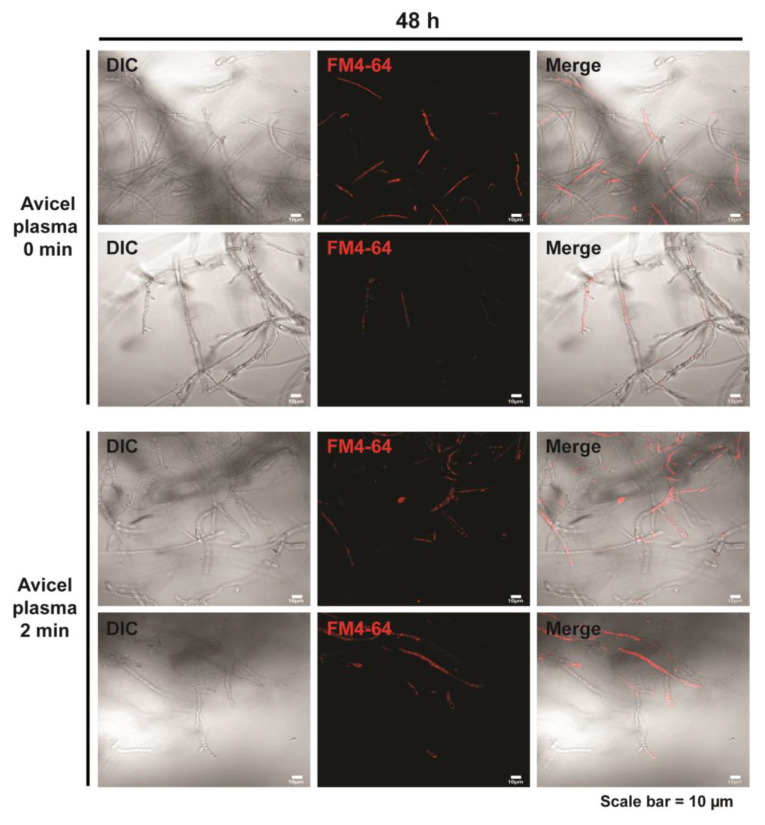
The vesicles in the fungal hyphae stained with FM4-64 48 h after plasma treatment. Fungal hyphae were treated in media containing Avicel with a plasma jet for 0 min or 2 min. Hyphae not treated with a plasma jet were used as controls. Two replicate pictures in each treatment are presented.

The level of cellulase production depends on gene expression and protein vesicle secretion capacity. Extracellular protein transport and release in filamentous fungi includes vesicle transport and secretion [9]. The secretion of extracellular proteins is generally thought to occur in the apical or sub-apical region of the hyphae [20,21]. We stained vesicles with the fluorescent dye FM4-64 48 h after a 2 min plasma treatment. A slight increase in fluorescence intensity was observed in the apical or sub-apical regions of the hyphae following plasma treatment compared to that observed in the apical or sub-apical areas of the untreated hyphae (Figure 5 and Figure S4a,b).

### 2.4. Plasma-Induced Enzyme Secretion Is Related to Elevated Level of Intracellular NO

**Figure 6:**
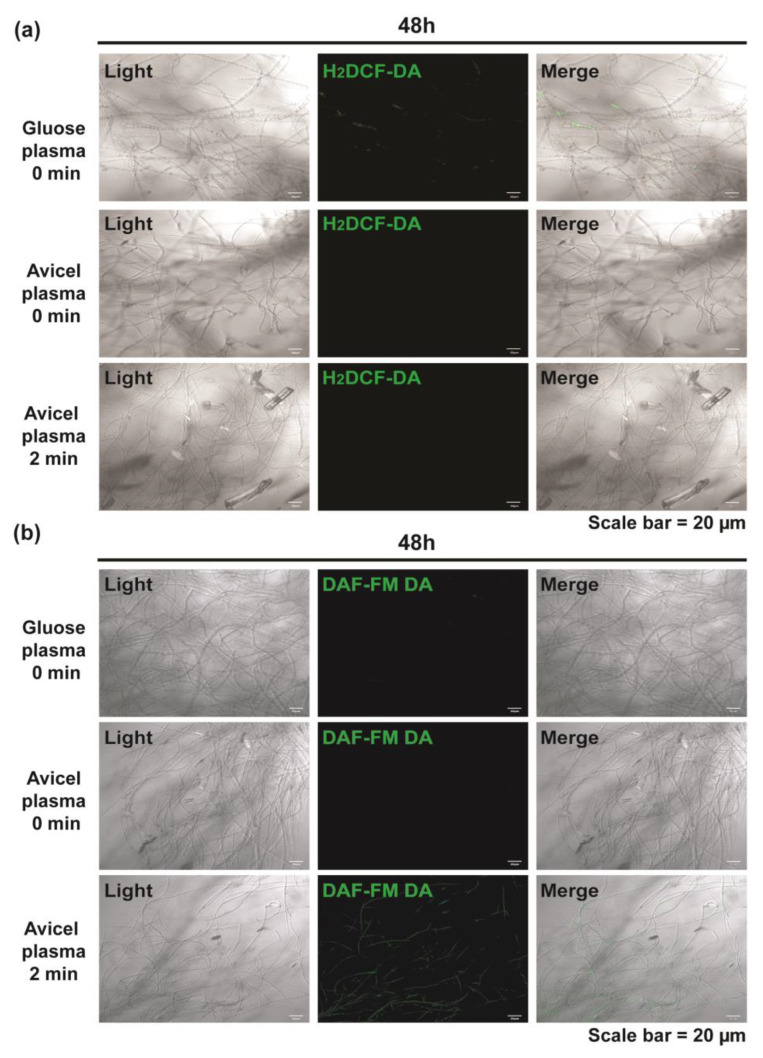
Intracellular ROS and NO stained with H_2_DCF-DA and DAF-FM DA, respectively, in fungal hyphae 48 h after plasma treatment for 0 min or 2 min. (**a**) Intracellular ROS detected using the fluorescent dye H_2_DCF-DA. (**b**) Intracellular NO detected using the fluorescent dye DAF-FM DA.

To identify the underlying mechanism(s) of plasma treatment, we first examined the levels of intracellular ROS and NO, as plasma has been shown to control the intracellular ROS and NO levels during physiological activities of various cells [22,23]. The intracellular ROS level, measured using 2′,7′-dichlorodihydrofluorescein diacetate (H_2_DCF-DA) after fungal hyphae were treated with plasma for 2 min and then incubated for 48 h, was not significantly different between the plasma-treated and untreated groups (Figure 6a and Figure S5a). Fluorescence was rarely detected in all samples (Figure 6a and Figure S5a). However, the intracellular NO level was significantly increased 48 h after plasma treatment in the presence of Avicel (Figure 6b and Figure S5b). Without plasma treatment, the intracellular ROS and NO levels were rarely elevated in fungal hyphae incubated in either the glucose or Avicel media, indicating that plasma played a major role in elevating intracellular NO level (Figure 6 and Figure S5).

**Figure 7:**
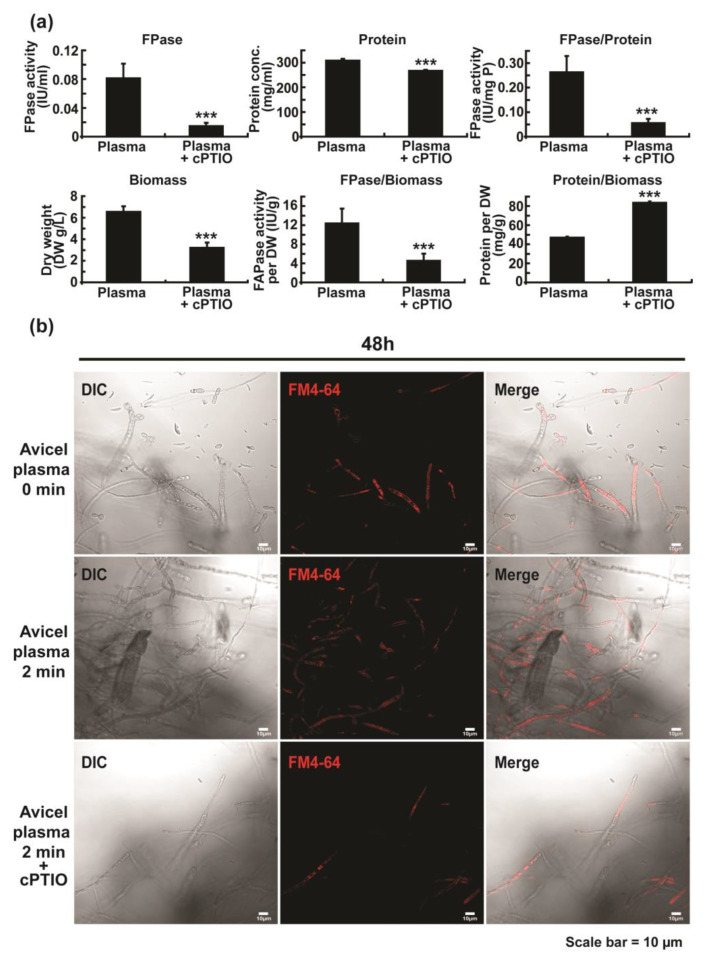
Effects of cPTIO on fungal cellulase production and vesicle accumulation. Fungal hyphae were treated with a plasma jet for 0 min or 2 min and the analysis was performed at 48 h after the addition of cPTIO. (**a**) Effects of cPTIO on total activity of cellulolytic enzymes, total protein concentration, activity of cellulase per mg of secreted protein, fungal biomass, activity of cellulolytic enzymes per g of biomass, and secreted protein per g of biomass. Each value represents the mean of 9–12 replicate measurements: *** *p* < 0.001. (**b**) Fungal hyphae vesicles stained with FM4-64 48 h after the addition of cPTIO.

To clarify the relationship between NO and cellulase production, the NO scavenger cPTIO was used. We discovered that the total activity of cellulolytic enzymes, total protein concentration, and specific cellulase activity in media were significantly reduced following the addition of cPTIO (Figure 7a). Furthermore, the cPTIO treatment significantly decreased the biomass of fungal hyphae (Figure 7a). This indicates that inhibited fungal growth via cPTIO may affect enzyme production. To test this, we calculated the relative enzyme activity or protein amount compared to fungal biomass. Total enzyme activity estimated per unit of fungal biomass (FPase/Biomass) was still significantly decreased following the addition of cPTIO, although the total protein per unit of biomass (Protein/Biomass) was significantly increased (Figure 7a). This indicates that reduction in enzyme activity may result from the removal of intracellular NO by cPTIO. We also examined the effect of cPTIO on vesicle accumulation in fungal hyphae. The fluorescence intensity of the apical or sub-apical region of the hyphae significantly decreased after the addition of cPTIO (Figure 7b and Figure S6).

### 2.5. Plasma Induces Intracellular Calcium Accumulation and Membrane Depolarization

**Figure 8:**
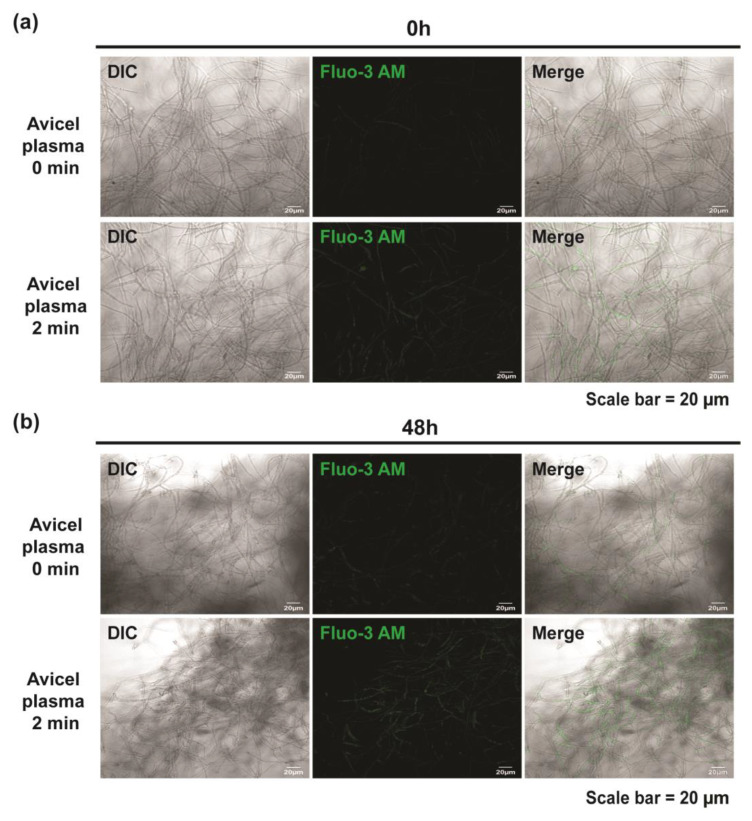
Assay for intracellular Ca^2+^ levels using Fluo-3 AM in the fungal hyphae at 0 (**a**) and 48 h (**b**) after plasma treatment for 0 or 2 min in Avicel-containing media.

In mammalian nerve cells, an increase in intracellular calcium triggers the activation of the nitric oxide synthase (NOS), resulting in an increase in intracellular NO levels [24]. In addition, numerous studies have shown that Ca^2+^ signaling plays an important role in vesicle transport and secretion [25,26]. We measured the intracellular Ca^2+^ in fungal hyphae using a fluorescent dye, Fluo-3 AM, following plasma treatment. The results showed that plasma treatments could significantly increase the intracellular calcium level (Figure 8 and Figure S7). The fluorescence level in fungal hyphae cultured in Avicel media was higher right after plasma treatment (0 h) (Figure 8a and Figure S7a). The increased fluorescence level was maintained in plasma-treated samples after 48 h (Figure 8b and Figure S7b). Moreover, we found no significant changes in intracellular calcium levels after the addition of the NO scavenger cPTIO (Figure S7c).

**Figure 9:**
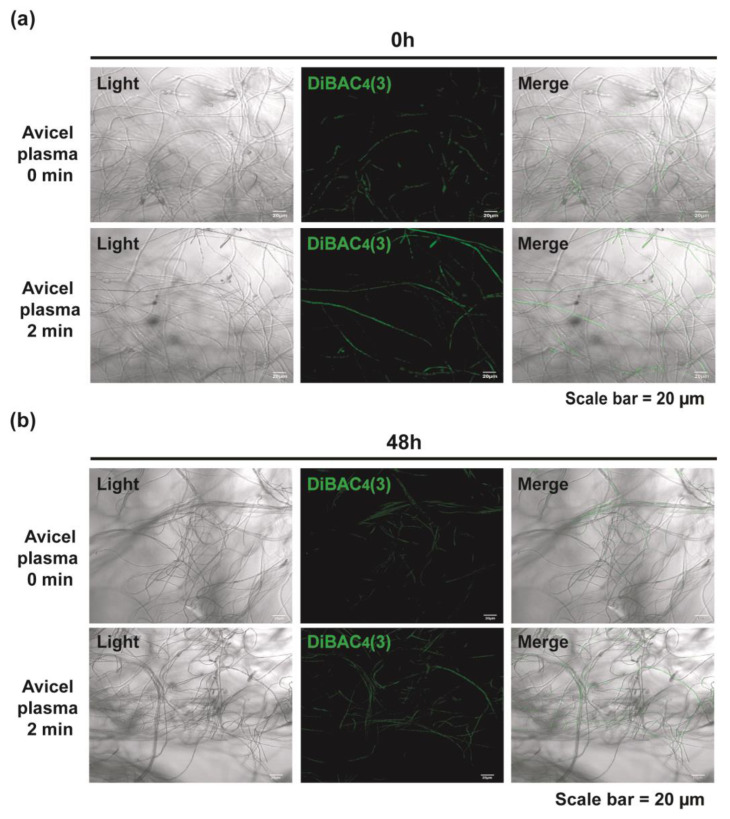
Assay for membrane potential using DiBAC_4_(3) in fungal hyphae at 0 (**a**) and 48 h (**b**) after plasma treatment for 2 min in Avicel-containing media.

Within the synaptic area of nerve cells, an influx of calcium ions into the cell is induced by membrane depolarization, and intracellular calcium accumulation in turn activates the accumulation of secretory vesicles near the membrane surface, leading to the secretion of neurotransmitters into the synaptic area [27]. We investigated the membrane depolarization of fungal hyphae using a fluorescent dye DiBAC_4_(3) to identify the relationship between membrane depolarization and intracellular Ca^2+^ level. Slightly increased fluorescence levels were observed in the plasma-treated fungal hyphae compared to those observed in the untreated samples after 0 (right after plasma treatment) and 48 h; this indicated that membrane depolarization was elevated right after (0 h) and 48 h after plasma treatment (Figure 9 and Figure S8).

## 3. Discussion

Previous studies have demonstrated that plasma has enhancing effects on wound healing, cell differentiation, seed germination, and growth [28,29]. However, plasma applications for promoting the production of industrially useful microbial enzymes have rarely been studied. Although previous studies have been conducted using plasma as a mutagenesis tool to generate fungal mutants with a high yield of enzymes or materials, the effects on the cellular process of enzyme production have rarely been explored (see [11]). Our study provides a series of experimental evidence showing that plasma can be a useful tool for promoting fungal enzyme production by elevating the level of enzyme gene expression and protein secretion. We quantified the promoting effect of plasma jet treatments based on cellulase activity and total protein concentration for the first time, and thereby provide a solid theoretical basis for the industrial application of plasma treatments. Our results are not mutation-induced because the plasma dose used in our study is much lower compared to those used in mutagenesis studies (see [30]). Furthermore, almost 100% of fungal spores survived following our plasma treatment, while over 90% of fungi were dead, and only those surviving were selected over several generations in mutagenesis studies [30]. We also observed that the increase in enzyme activity in media after plasma treatment was slowed down over incubation time. This indicates that the enhancing effect of plasma may be temporary, and not inheritable (no genetic change). In this study, plasma enhanced the production of cellulolytic enzymes in *N. crassa*, which are different enzymes in a different fungal species from those presented in our previous study [14]. This indicates that plasma treatments might represent universal tools for promoting the production of industrially useful enzymes in fungi. Furthermore, we applied plasma to fungal spores in our previous study [31] and fungal hyphae (24 h-old) in this study. This provides a possibility that plasma treatment during two different developmental stages of fungi can enhance the fungal enzyme production.

However, our study pointed out several aspects that need to be considered for plasma to be a reliable tool for activating enzyme production in fungi. First, the improvement in the secreted cellulolytic enzyme levels by plasma was modest, approximately 1.1~1.2-fold, although it was significant. The level of transcription of four major cellulolytic enzyme genes was increased 1.3~17-fold in Avicel media by plasma. If mRNAs of the four enzymes are translated into proteins, and proteins are completely secreted into media, the1.1~1.2-fold increase in secreted enzymes level (measured as the enzyme activity in media) is lower than expected. This indicates that the enzyme secretion process may not be considerably affected by the plasma treatment. Further investigation may be necessary to reveal the plasma effects on the interaction between enzyme gene expression and the secretory pathway. Another important aspect was that the plasma-mediated improvement was dramatic in Avicel media. Although the plasma treatment increased the mRNA levels of enzyme genes in the non-inducing glucose media, the increased level (1.2~2.2-fold) was slight compared to that in Avicel media. Avicel (microcrystalline cellulose) is considered an efficient inducer for cellulase production [19]. Our results suggest that plasma might regulate the process through which Avicel induces cellulase production. In future applications, plasma can be used as an auxiliary to improve enzyme production. For example, it can be used in combination with inducers to further improve enzyme production.

Regarding the mechanism(s) of plasma-mediated enhancement of enzyme production, our results suggest that the elevation in intracellular NO and Ca^2+^ levels following plasma treatment may play a major role in the enhancement of enzyme production. ROS, NO, and Ca^2+^ are generally recognized as intracellular signal molecules. Exogenous signals are transmitted into the cell via receptors on the plasma membrane. The receptors transmit the exogenous signals downward, and these signals are often converted into endogenous signals inside cells such as ROS, NO, and Ca^2+^ [32,33]. In the fungal model species *Saccharomyces cerevisiae*, arsenic treatment can induce the production of intracellular ROS and Ca^2+^ signals [34]. Exogenous copper ions also induce a substantial increase in intracellular NO production in *S. cerevisiae* [35]. Similarly, we observed an increase in the levels of intracellular NO and Ca^2+^ after plasma treatment. This indicates that the plasma jet, an exogenous signal, triggered the generation of endogenous signals, NO and Ca^2+^, and these second messengers are involved in enhancing the production of cellulases, probably by influencing the secretory process (NO and Ca^2+^ levels were measured after 48 h). The protein secretion pathway in filamentous fungi includes cellular trafficking of secretory vesicles containing proteins, and secretion occurs at the apical and sub-apical area of hyphae [9,36]. Therefore, tracing secretory vesicles might allow for the measurement of protein secretion levels. By staining vesicles, we demonstrated that the secretory vesicle output in fungal hyphae was reduced when intracellular NO was removed by cPTIO after plasma treatment. We previously observed that the amylase production capacity of *Aspergillus oryzae* (fungus) was significantly enhanced when the NO donor SNP was added [31]. These findings support our hypothesis that plasma may elevate the generation of intracellular NO, which in turn activates the secretion of fungal enzymes.

Additionally, Ca^2+^ has a regulatory effect on the plasma mediated enhancement in cellulase production, as demonstrated in our data. We showed that Ca^2+^ was accumulated inside fungal cells upon plasma treatment. In *Trichoderma reesei*, Ca^2+^ promotes the transcription of cellulase genes such as *cbh1* and *cbh2* by activating the calmodulin-Crz1 signaling pathway [10,37,38]. Nevertheless, it is not clear whether the increase in Ca^2+^ levels within cells affected the expression of cellulolytic enzyme genes because we measured the Ca^2+^ level 48 h after the plasma treatment, which was far beyond the gene expression time window. Nonetheless, the increased intracellular Ca^2+^ level seems to enhance the enzyme secretion process. This is in line with previous studies that showed that Ca^2+^ signaling plays an important role in the process of vesicle transport and secretion [25]. 

An increase in Ca^2+^ level is likely to be associated with membrane depolarization after plasma treatment, since we showed that the level of cell membrane depolarization was higher in plasma-treated than in non-treated fungal hyphae. Previously, we also observed that plasma increases membrane depolarization in *A. oryzae* [31]. When a plasma jet is applied, it will ionize the air, resulting in the elevation of anion concentration in the culture media. As a result, the fungal hyphal cell membrane is depolarized, and the depolarization of the cell membrane may lead to abnormal activation of Ca^2+^ channels and Ca^2+^ pumps on the membrane, leading to the entry of extracellular Ca^2+^ into the cytoplasm. A potential anion with an elevated concentration may be nitrate (NO_3_^-^). Our previous study showed that large amounts of NO_3_^-^ are produced in plasma-treated solutions and are involved in promoting enzyme secretion [31]. In plants, NO_3_^-^ can lead to the depolarization of cell membranes during plant root uptake [39,40]. Nitrate-dependent depolarization occurs rapidly at a pH of 5.7 and a concentration of 2 mM, which can be detected after approximately 2 min in *Lemna gibba cells* [41]. In our study, fungal hyphae were transferred to new media right after plasma treatment; thus, the fungal hyphae were exposed to plasma-induced NO_3_^-^ in media within a very short time. Although we did not measure the NO_3_^-^ concentration, the NOx level in media was slightly increased after plasma treatment (Figure 1c). However, it is highly plausible that the depolarization of the cell membrane after plasma treatment may be nitrate-dependent; further research is necessary to clarify this. 

Although plasma treatments can elevate the levels of intracellular NO and Ca^2+^, the relationship between intracellular NO and Ca^2+^ upon plasma treatment is not clear. Several studies on mammalian cells showed that intracellular calcium enhances NO production by activating NOS [24,42,43]. Our data showed that NO scavenging did not affect the intracellular Ca^2+^ level after plasma treatment. This suggests that plasma may induce membrane depolarization, which in turn increases the intracellular level of Ca^2+^, resulting in the activation of NO production. On the other hand, plasma may directly induce the increase in intracellular NO level through a particular mechanism, which is a subject for future study. In both cases, how the elevated amount of intracellular NO is involved in the activation of enzyme production is another subject needed for further analysis. Our hypothetical mechanism is that the increased level of NO inside the cell may be involved in regulating the cellular trafficking of secretory enzymes, since we have observed the decrease in number of vesicles (probably containing enzymes) after NO scavenging. 

We also discovered that the promoting effect of plasma was inhibited with longer and more frequent plasma treatments. This suggests that high-intensity plasma treatments may cause the inhibitory effects on fungal cellular processes, which may be related to the concentration of ROS and NOx produced by plasma. With the increase in the number and intensity of the plasma treatments, the ROS and NOx concentrations in the media increase continuously, which will induce cell death [44]. Possibly, there is a plasma dose threshold for the activation of the enzyme production. The plasma dose can be determined by the electrical power of the plasma device, treatment time and number, level of generated reactive species, or mixture of all (because these factors correlate with each other). In our study, we observed the inhibition effect of the increased number and time of plasma treatment, and therefore, plasma dose refers to the treatment time and number. The elevation of treatment time and number might contribute to increasing the level of ROS and NOx, although we did not measure the concentration in all cases. Further analysis and fine-tuning the plasma dose are crucial for obtaining the maximum promoting effect of plasma on fungal enzyme production.

Because air plasma can have a relatively high power and this may lead to the heating of the plasma gas, the possibility of thermal effects on fungal enzyme production can be considered in our study. We used the inverter, operated in dimming mode, to generate plasma, and plasma was periodically switched on and off (13 ms and 94 ms on-time and off-time, respectively). This periodic switching-off of plasma may prevent the over- heating of the electrodes, as well as gas heating. In addition, the distance between the end of the plasma device and the surface of the sample media was 20 mm, the volume of the media was high (30 mL), and the gas flow rate was high (1.5 lpm). Therefore, the warming-up of the sample after plasma treatment may only rarely occur, not supporting the possibility of a thermal effect. The effect of an electric field may nonexistent, or very trivial, because the inner electrode was enclosed by an outer metal ground electrode in our plasma device, and fungal hyphae were treated in the submerged state in medium, far away from the plasma device (20 mm). 

## 4. Materials and Methods

### 4.1. Fungal Strain and Culture Condition

The fungus used in this study was *Neurospora crassa*, a model filamentous fungus. *N. crassa* strain (strain name, ORS-SL6a; mating type, mat a; FGSC number, 4200) was obtained from the Fungal Genetics Stock Center (FGSC, Manhattan, KS, USA). The fungus was maintained on Vogel’s Minimal (VM) agar plates. To obtain conidia, the fungus was inoculated onto VM agar media and cultured at 30 °C in darkness for 2 days and then at 25 °C in light for 12 days. For liquid culture, VM liquid inoculated with fungal spores (10^6^/mL) was incubated at 25 °C under constant light with shaking at 200 rpm.

### 4.2. Plasma Treatment

A non-thermal atmospheric pressure plasma jet (Figure 1a) was used to treat fungal hyphae in this study. Plasma jet equipment was provided by the Plasma Bioscience Research Center (PBRC) from Kwangwoon University (Seoul, Korea). The electrode configuration and electrical properties of the plasma device have been comprehensively described in a previous study [45]. A dielectric glass tube (3.3 mm inner diameter and 5 mm outer diameter) containing a needle electrode (1.7 mm inner diameter) was placed inside a ground metal electrode (6 mm inner diameter and 10 mm outer diameter) with a gap of 1 mm between the dielectric glass end and the tip of the ground metal electrode. Air (1.5 lpm) was used as the feeding gas. The inverter (operated in dimming mode) voltage was applied to generate the plasma jet: ~0.68 kV applied voltage (rms), ~77 mA current (rms) of the discharge, ~83 kHz frequency, 13 ms and 94 ms on-time and off-time of the device, respectively (~12% duty percentage), and 0.37 W dissipated power [45]. The optical emission spectra of plasma discharge were also well characterized in the previous study [45]. Emissions of NOγ and OH radicals were detected in the range of 200–280 nm and 306–309 nm, respectively. Several emissions from the nitrogen second positive system and nitrogen first negative system were detected, as well as those from atomic nitrogen (742 nm, 822 nm, and 868 nm) and atomic oxygen (777 nm and 845 nm).

For plasma treatment, fungal mycelia were first prepared. Spores of *N. crassa* were inoculated into 30 mL VM liquid with 2% (*w*/*v*) glucose (1 × 10^6^ spores/mL) placed in a glass Erlenmeyer flask (85 mm diameter, 140 mm height, 34 mm neck diameter), and the flask was incubated at 25 °C under constant light with shaking (200 rpm) for 24 h. Next, the air plasma jet device was placed inside the flask (20 mm distance between the end of metal electrode and the surface of VM liquid), and then plasma jet was applied for 2 and 5 min (Figure 1a). Because the plasma plume was less than 1 cm in length, it did not touch the surface of medium. Fungal mycelia were collected by filtration through 2 layers of Miracloth (EMD Millipore, Burlington, MA, USA) and then washed with deionized water. Fungal mycelia were resuspended in 30 mL new VM containing 2% (*w*/*v*) glucose (Duksan, Seoul, Korea) or 2% (*w*/*v*) Avicel (Avicel PH-101, Sigma-Aldrich, St. Louis, MI, USA) and incubated at 25 °C under constant light with shaking (200 rpm) for the indicated time. 

For scavenging the intracellular NO, fungal mycelia were prepared and treated with a plasma jet, as described above. Next, plasma treated fungal mycelia were collected and resuspended in a new VM containing 2% (*w*/*v*) Avicel and 10 mM cPTIO (Calbiochem, San Diego, CA, USA).

### 4.3. Measurement of H_2_O_2_, NO_X_ Levels, ORP, and pH in the Media 

To measure H_2_O_2_ and NO_X_ levels in media, only culture media was collected by spinning down fungal mycelia and collecting the supernatant immediately after the plasma treatment. The H_2_O_2_ and NO_X_ levels were measured using an Amplex^TM^ Red Hydrogen Peroxide/Peroxidase Assay Kit (Molecular Probes, Eugene, OR, USA) and a QuantiChrom^TM^ Nitric Oxide Assay Kit (BioAssay Systems, Hayward, CA, USA), respectively, according to the manufacturer’s instructions. The oxidation reduction potential (ORP) was measured using an ExStik^TM^ Model RE300 waterproof ORP meter (Extech, Nashua, NH, USA), and the pH was measured using a portable pH meter (Oakton Instruments, Vernonb Hills, IL, USA).

### 4.4. Assay for Determining the Activities of Cellulolytic Enzymes

To measure protein concentration and cellulase activity in the media, culture tubes were centrifuged at 2390× *g* for 10 min, and then supernatants were harvested at 24, 48, and 72 h after the plasma treatment. The harvested supernatants were stored at 4 °C. After all samples were collected, enzyme activity and total protein concentration were measured. 

Total protein concentration was determined using the Bradford protein assay kit (Bio-Rad, Hercules, CA, USA) following the manufacturer’s protocol. Total activity of cellulolytic enzymes (FPase) was determined by the rate of degradation of the filter paper, as described previously [46]. Briefly, a reaction mixture containing Whatman filter paper no. 1. (substrate; Cytiva, Marlborough, MA, USA), 30 μL of 0.1 M acetate buffer (pH 5.6), and 30 μL of culture supernatant was incubated at 50 °C for 30 min. Liberated reducing sugar levels (a product of the enzymatic reaction) were measured by adding 120 μL of 3,5-dinitrisalicylic aid (DNS; Sigma-Aldrich, St. Louis, MI, USA) in the reaction mixture and then boiling for 10 min. After 720 μL of deionized water was added to the reaction mixture, the solution absorbance was measured at 540 nm using a microplate reader (Biotek, Winooski, VT, USA). One unit of enzymatic activity was defined as the amount of enzyme capable of producing 1 μM of reducing sugars from the appropriate substrates per min.

### 4.5. Quantitative Real-Time PCR Analysis

Quantitative real-time PCR was used to assess the gene expression levels. Fungal mycelia were collected by filtration through the Miracloth (EMD Millipore) at 0, 2, and 4 h after plasma treatment, and immediately frozen in liquid nitrogen and stored at −80 °C until analysis. Total RNA was extracted from fungal mycelia using RNAiso Plus (TaKaRa Bio, Shiga, Japan), and cDNA was synthesized using the ReverTra Ace qPCR RT Master Mix with gDNA Remover (Toyobo, Osaka, Japan), following the manufacturer’s protocols. Real-time PCR was performed using the iQ SYBR Green Supermix (Bio-Rad) and CFX 96^TM^ real time Instrument (Bio-Rad), following the manufacturer’s instructions. The primer sequences [16] are listed in Table 1. β-actin was used as a reference gene. The mRNA levels for each enzyme were normalized to the reference gene (β-actin): mRNA level of enzyme gene = 2^−∆∆Ct^, where ∆∆Ct = (Ct _target_ − Ct _reference_) plasma treatment-(Ct _target_ − Ct _reference_) control [47]. 

### 4.6. Protein Gel Electrophoresis

Culture supernatants were collected at 24, 48, and 72 h after plasma treatment through centrifugation at 2390× *g* for 10 min. Sample buffer (5×) was added to the culture supernatant, and the mixture was boiled for 5 min. A total of 20 μL of the solution was applied onto sodium dodecyl sulfate (SDS)-polyacrylamide (12%) gel. The gel was run at 225 V for approximately 1 h. After electrophoresis, the gel was stained overnight using Coomassie Blue R-250 (Bio-Rad, Hercules, CA, USA), and then the background was washed out with Destain solution. Lastly, the gel was imaged using ChemiDoc^TM^ MP imaging system (Bio-Rad) and analyzed using ImageJ software version 1.52a (National Institute of Health, Bethesda, MD, USA).

### 4.7. Analysis for Intracellular ROS and RNS Levels

Fungal mycelia were harvested at the indicated times and washed twice with 1× phosphate-buffered saline (PBS). To detect intracellular ROS, fungal mycelia were incubated with 20 μM of H_2_DCF-DA (Themo Fisher, Waltham, MA, USA) at 25 °C for 1 h. To detect intracellular NO, fungal mycelia were incubated with 20 μM of 4-Amino-5-Methylamino-2′,7′-Difluorofluorescein Diacetate (DAF-FM DA, Themo Fisher, Waltham, MA, USA) at 25 °C for 1 h. After incubation, fungal mycelia were washed with 1× PBS at least 3 times and examined under a FV-100 MPE spectra confocal laser scanning microscope (Olympus Corporation, Tokyo, Japan).

### 4.8. Determination of Membrane Potential, Intracellular Ca^2+^, and Secretory Vesicles

Membrane potential, intracellular Ca^2+^, and secretory vesicles were analyzed as previously described [31,44]. Fungal mycelia were harvested at 0 and 48 h after plasma treatment and washed twice with 1× PBS. For measuring membrane potential, fungal mycelia were incubated with 50 μg/mL of Bis-(1,3-dibutylbarbituric acid) trimethine oxonol (DiBAC4(3); Invitrogen, Carlsbad, CA, USA) at 4 °C in the dark for 1 h. For detecting Ca^2+^, fungal mycelia were incubated with 5 μM of Fluo3-AM (Invitrogen) at 25 °C in the dark for 1 h. For staining vesicles, fungal mycelia were incubated with 25 μM of FM4 4–64 (Invitrogen) at 30 °C in the dark for 30 min. All stained and labelled samples were analyzed and imaged using a FV-100 MPE spectra confocal laser scanning microscope (Olympus Corporation).

### 4.9. Statistical Analysis

All data are presented as the mean ± standard deviation (SD) from at least six replicates. Paired Student’s *t*-tests and two-way analysis of variance were performed, followed by Tukey’s post hoc test. A *p*-value < 0.05 was considered to reflect a statistically significant difference. SPSS Statistics Software, version 25 (IBM, Chicago, IL, USA) was used for statistical analysis.

## 5. Conclusions

Our results demonstrate that plasma treatment can promote the transcription and secretion of *N. crassa* cellulolytic enzymes in the presence of Avicel (induction condition). A hypothetical mechanism for this is that plasma can induce membrane depolarization, which in turn increases the intracellular Ca^2+^ level, thereby activating NO production. The increased levels of Ca^2+^ and intracellular NO may have acted as signals for activating enzyme expression and secretion.

The bioproduction of industrially useful enzymes by microorganisms requires a high level of enzyme intracellular expression and a high efficiency of extracellular enzyme secretion. If one of these two processes is inefficient, it could cause a bottleneck for large-scale enzyme production. Fungi are considered a good resource for the industrial-scale production of useful enzymes, and our study provides additional experimental evidence showing that plasma treatments might represent a potential tool for making fungi more reliable producers of industrial enzymes by improving enzyme secretion and/or enzyme gene expression. Interestingly, our study also shows that plasma can be used as an auxiliary tool, along with inducers (Avicel, in our study), to increase the efficiency of the inducer. Nevertheless, further research investigating the fine-tuning of plasma treatment conditions for obtaining maximum efficiency of enzyme production, as well as deciphering the detailed mechanism(s) underlying plasma-mediated enhancement, is needed. 

## Figures and Tables

**Table 1:** List of primers used in qRT-PCR [16].

Genes	Primer Sequences
*actin*	Forward- 5′-TGA TCT TAC CGA CTA CCT-3′Reverse- 5′-CAG AGC TTC TCC TTG ATG-3′
*cbh-1*	Forward- 5′-ATC TGG GAA GCG AAC AAA G-3′Reverse- 5′-TAG CGG TCG TCG GAA TAG-3′
*gh6-2*	Forward- 5′-CCC ATC ACC ACT ACT ACC-3′Reverse- 5′-CCA GCC CTG AAC ACC AAG-3′
*gh5-1*	Forward- 5′- GAG TTC ACA TTC CCT GAC A-3′Reverse- 5′-CGA AGC CAA CAC GGA AGA-3′
*gh3-4*	Forward- 5′- AAC AAG GTC AAC GGT ACG TGG-3′Reverse- 5′-TCG TCA TAT CCA TAC CAC TGT TTG-3′

## Supplementary Materials

The following are available online at https://www.mdpi.com/article/10.3390/ijms23126668/s1.
