# Supplementary material for: Plasma Promotes Fungal Cellulase Production by Regulating the Levels of Intracellular NO and Ca2+
Source: Int J Mol Sci. 2022 Jun 15;23(12):6668. doi: 10.3390/ijms23126668 (PMC9223429; doi:10.3390/ijms23126668)
Supplement: Supplementary file 1 [file ijms-23-06668-s001.zip › ijms-1736739-supplementary.pdf]

Supplementary Information

Plasma promotes fungal cellulase production by regulating the levels of intracellular NO and Ca^{2+}

Nan-Nan Yu[1], *Wirinthip Ketya* [1], *Eun-Ha Choi* [1,2] *and Gyungsoon Park*[1,2*]

[1]Department of Plasma-Bio Display and Plasma Bioscience Research Center, Kwangwoon University, Seoul, 01897, Korea

[2]Department of Electrical and Biological Physics, Kwangwoon University, Seoul, 01897, Korea

* Corresponding Author

Gyungsoon Park

Phone: +82-2-940-8324

Fax: +82-2-940-5664

Email: gyungp@kw.ac.kr

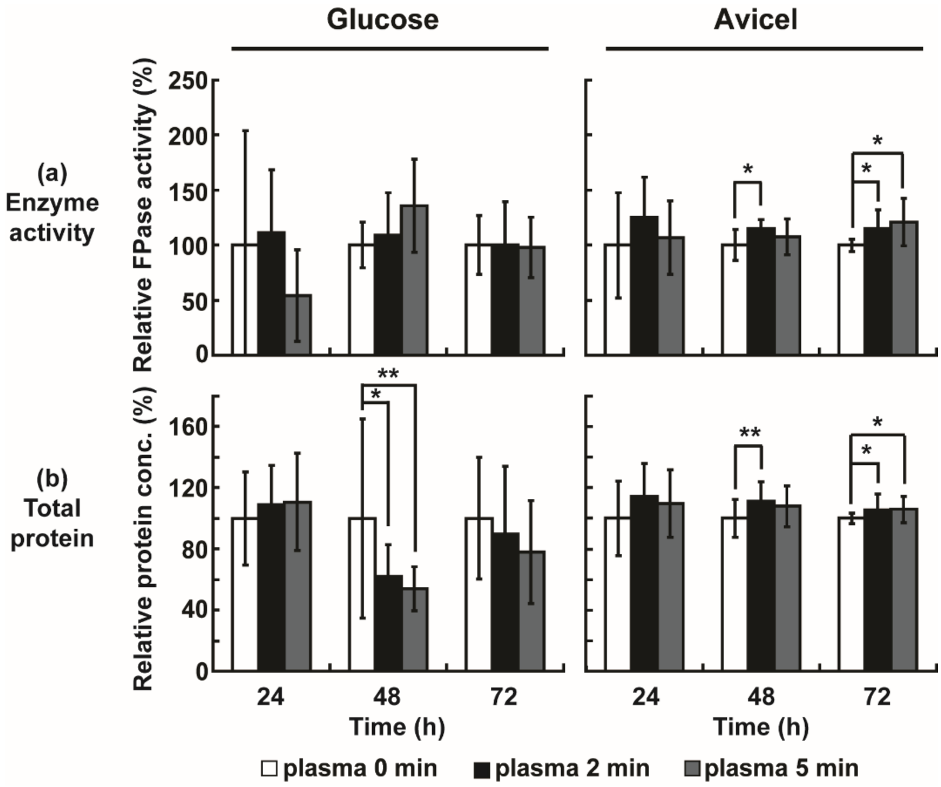

Supplementary Figure S1. **The relative filter paper enzyme activity and relative total protein concentration in media.** Relative filter paper enzyme activity (total activity of cellulolytic enzymes) (**a**) and relative total protein concentration (**b**) were calculated compared to those of no plasma treated groups. Each value is the mean of 9-12 replicate measurements: * $p < 0.05$, ** $p < 0.01$.

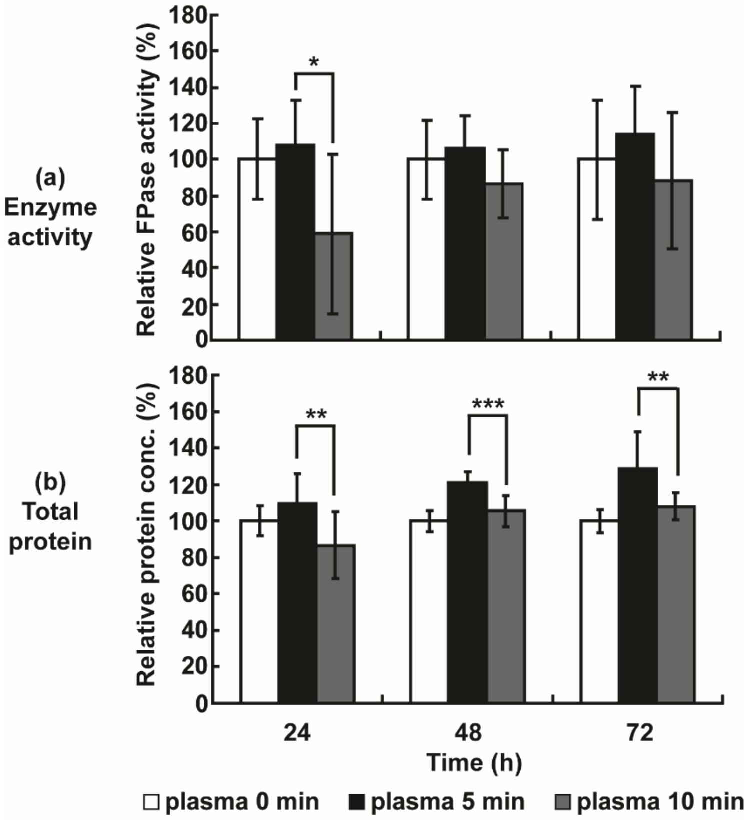

Supplementary Figure S2. Activity of total cellulolytic enzymes and total protein concentration in avicel media after plasma treatment. Relative filter paper enzyme activity (total activity of cellulolytic enzymes) (**a**) and relative total protein concentration (**b**), compared to those of no plasma treated group (0 min), were measured 24, 48, and 72 h after treatment with plasma for 5 and 10 min. Each value is the mean of 6 replicate measurements: * $p < 0.05$, ** $p < 0.01$, *** $p < 0.001$.

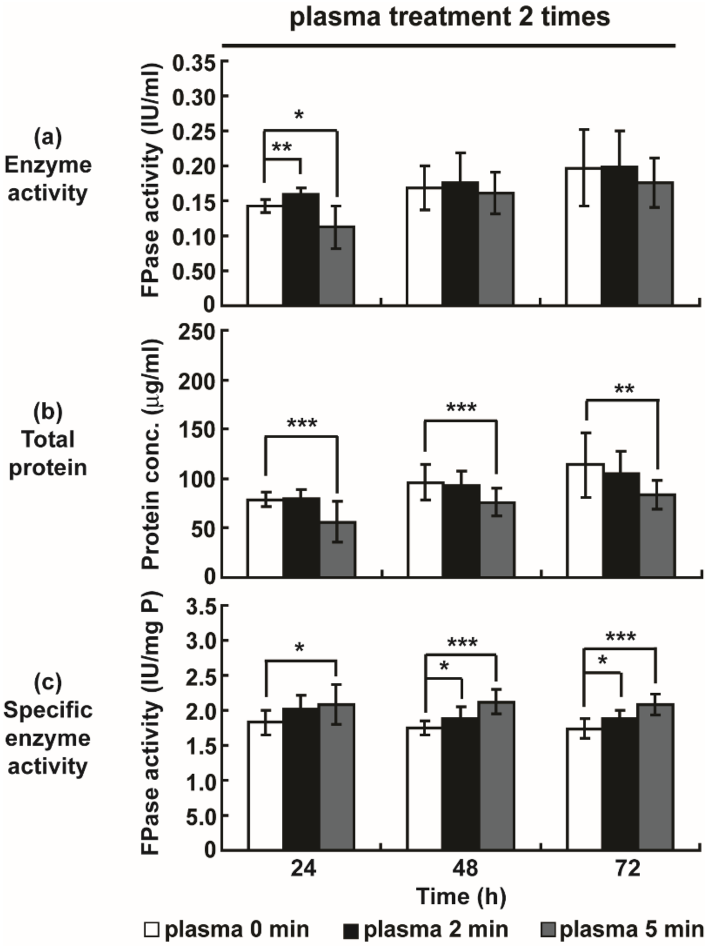

Supplementary Figure S3. Effect of 2-times plasma treatment on the activity of cellulolytic enzymes and total protein concentration in avicel media. Fungal hyphae grown for 24 h were treated with plasma jet (1st treatment) and then transferred to new media. Then, another treatment with plasma jet was performed (2nd treatment), and then fungal hyphae were transferred to new media. The media was harvested at 24h, 48h, 72h after last plasma treatment: (a) Filter paper enzyme (FPase) activity (total activity of cellulolytic enzymes); (b) Total protein concentration; (c) Specific enzyme activity. Each value the is mean of 9-12 replicate measurements: * $p < 0.05$, ** $p < 0.01$, *** $p < 0.001$.

(a)

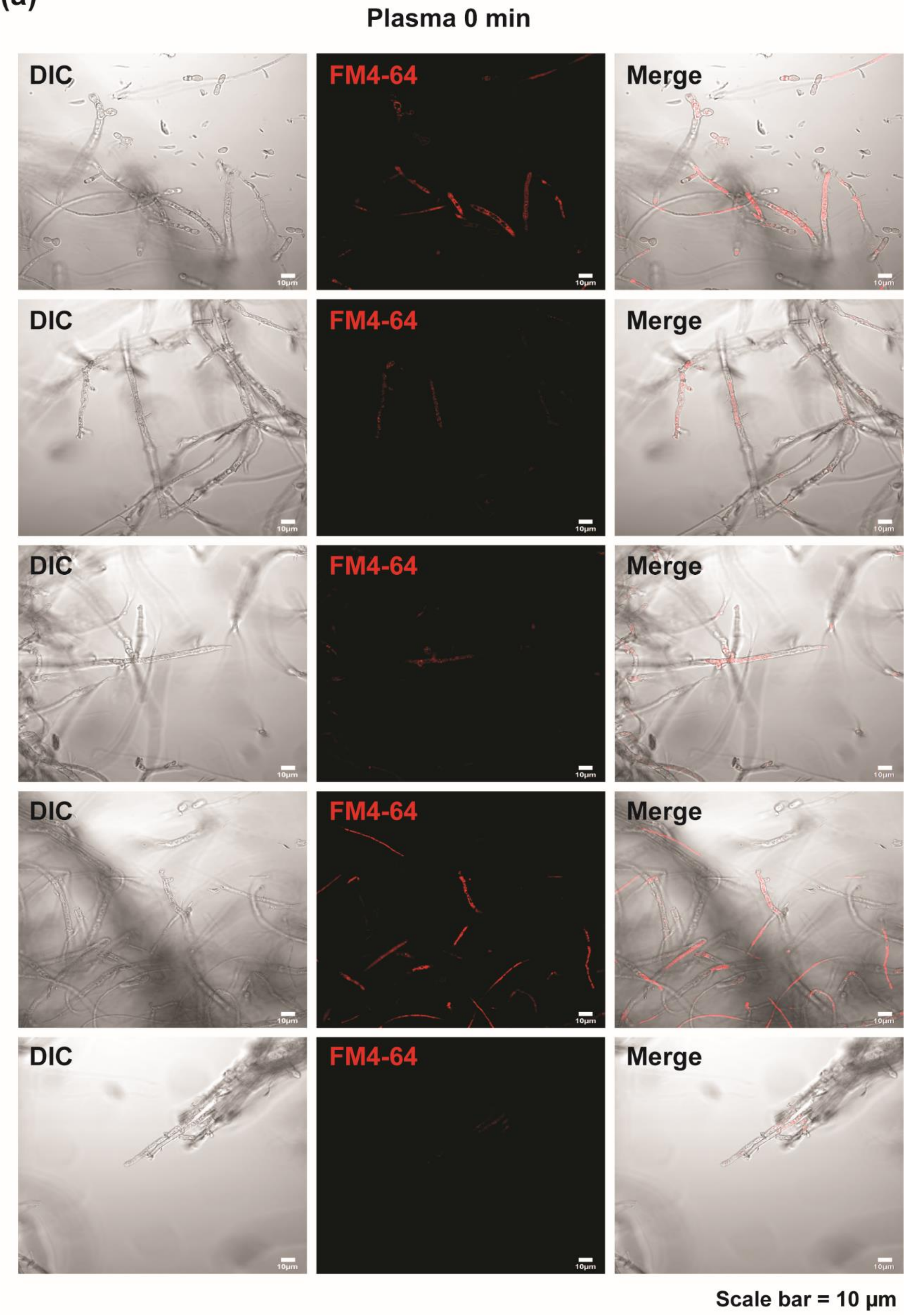

(b)

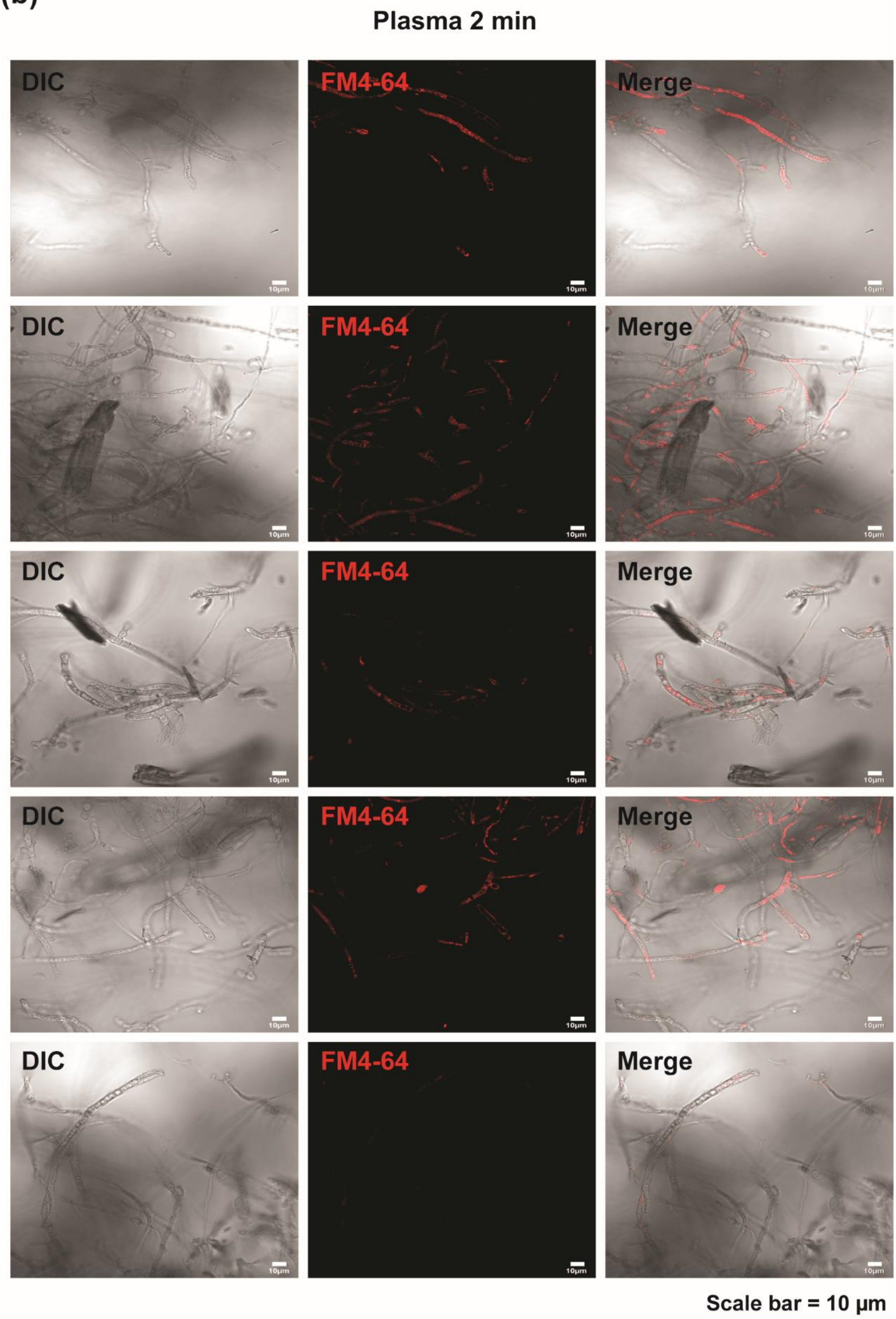
Plasma 2 min
DIC
FM4-64
Merge
DIC
FM4-64
Merge
DIC
FM4-64
Merge
DIC
FM4-64
Merge
DIC
FM4-64
Merge
Scale bar = 10 µm

Supplementary Figure S4. Vesicle staining in *N. crassa* hyphae after plasma treatment for 0 min (control) or 2 min. Fungal hyphae were stained with FM4-64 (red fluorescence) after 48 h of incubation in avicel media. Pictures of fungal hyphae in 5 different areas were taken. DIC; Differential Interference Contrast, FM4-64; fluorescence, Merge; combined image of DIC and fluorescence.

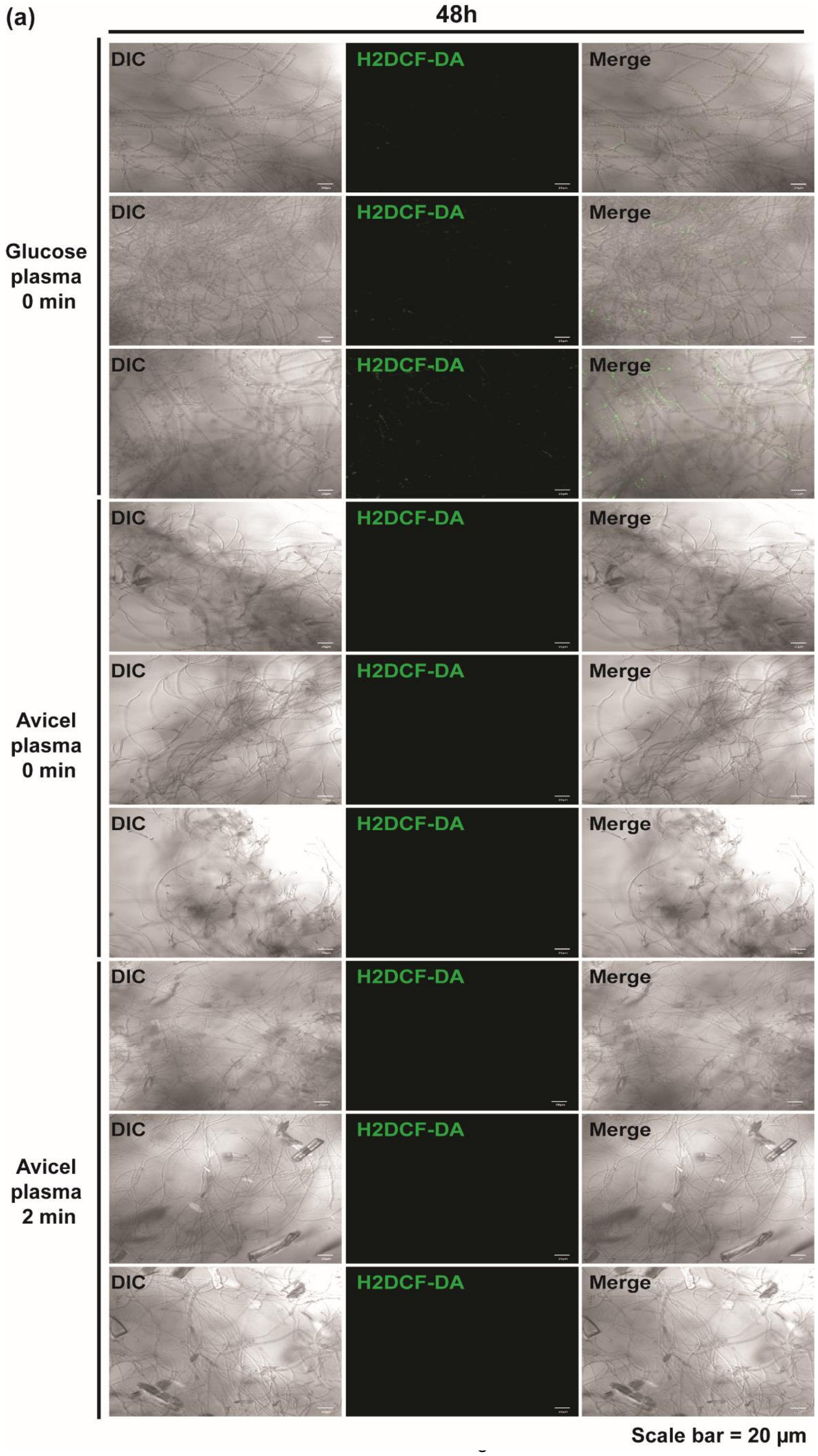
(a)
48h
DIC
H2DCF-DA
Merge
Glucose plasma 0 min
Avicel plasma 0 min
Avicel plasma 2 min
Scale bar = 20 µm

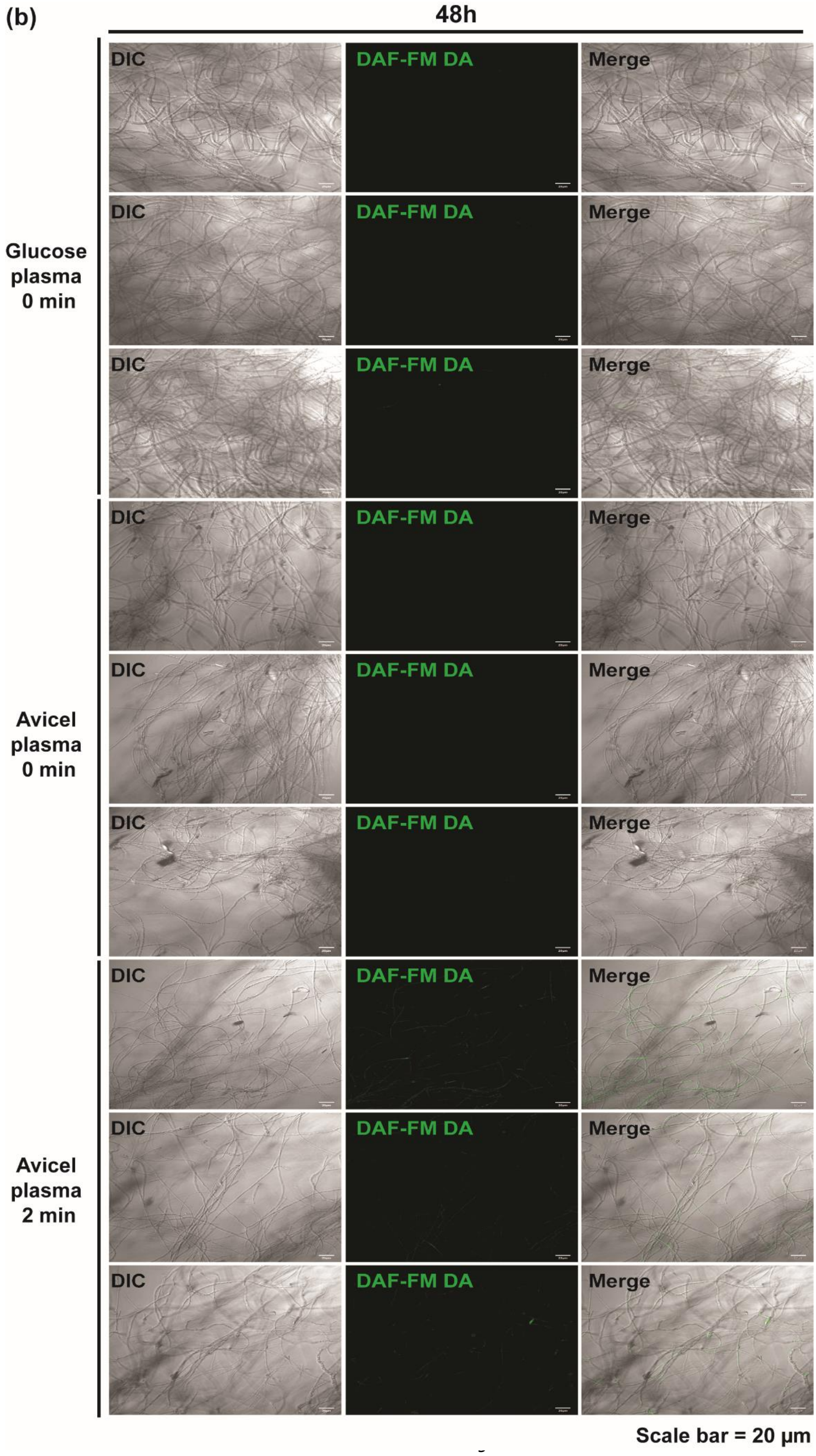
(b)
48h
DIC
DAF-FM DA
Merge
Glucose plasma 0 min
Avicel plasma 0 min
Avicel plasma 2 min
Scale bar = 20 μm

Supplementary Figure S5. Analysis of intracellular ROS and NO level in *N. crassa* hyphae after plasma treatment for 0 min (control) or 2 min. Fungal hyphae were stained with H_2DCF-DA (green fluorescence) for ROS (a) and DAF-FM DA (green fluorescence) for NO (b) after 48 h of incubation in avicel or glucose media. Pictures of fungal hyphae in 3 different areas were taken. DIC; Differential Interference Contrast, H_2DCF-DA or DAF-FM DA; fluorescence, Merge; combined image of DIC and fluorescence.

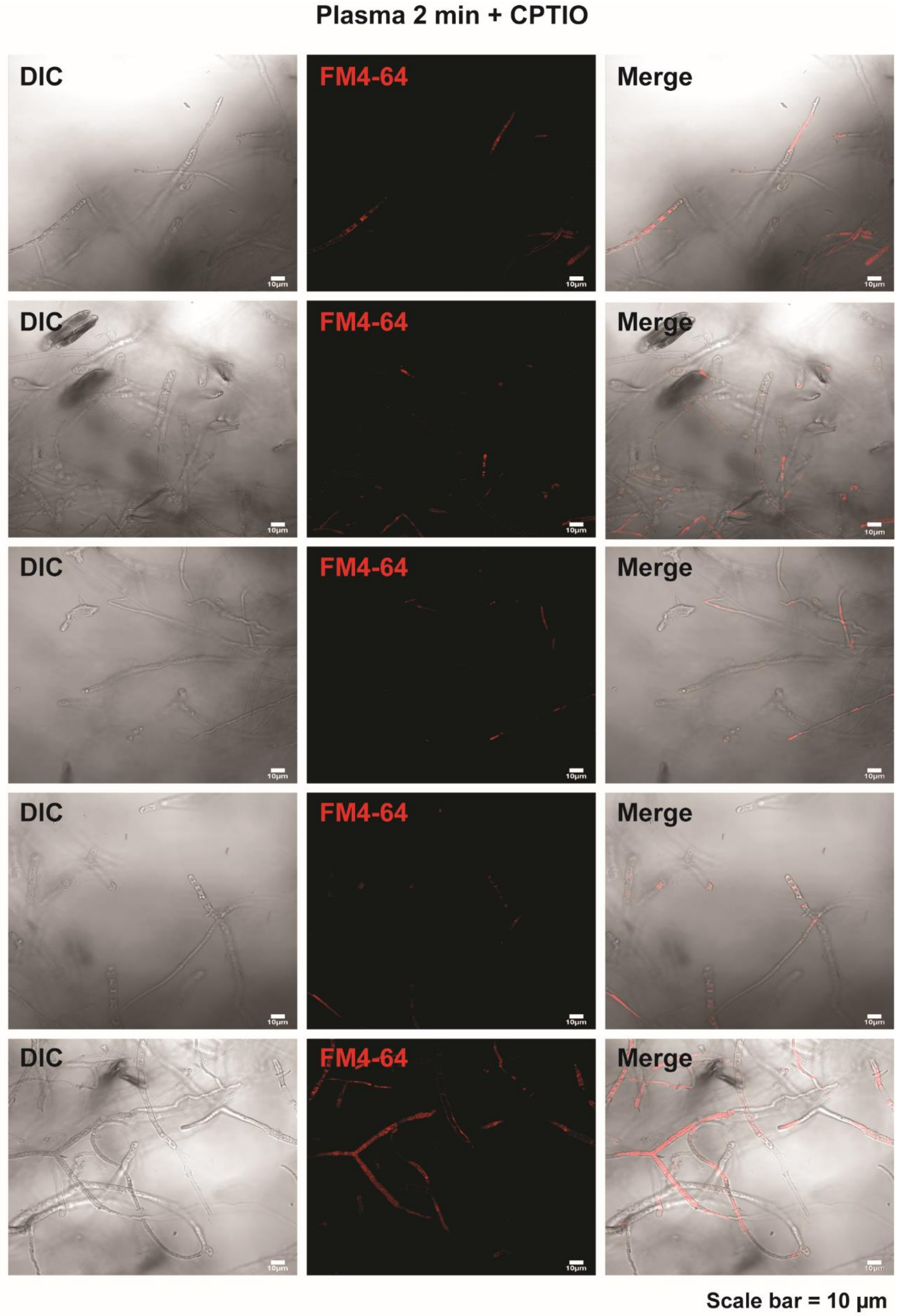

Supplementary Figure S6. Analysis of vesicles in *N. crassa* hyphae after 2 min of Plasma treatment with the addition of cPTIO. Mycelia were immediately transferred to avicel media

containing 10mM cPTIO after plasma treatment. After 48 h, fungal hyphae were stained with FM4-64 (red fluorescence). Pictures of fungal hyphae in 5 different areas were taken. DIC; Differential Interference Contrast, FM4-64; fluorescence, Merge; combined image of DIC and fluorescence.

(a)

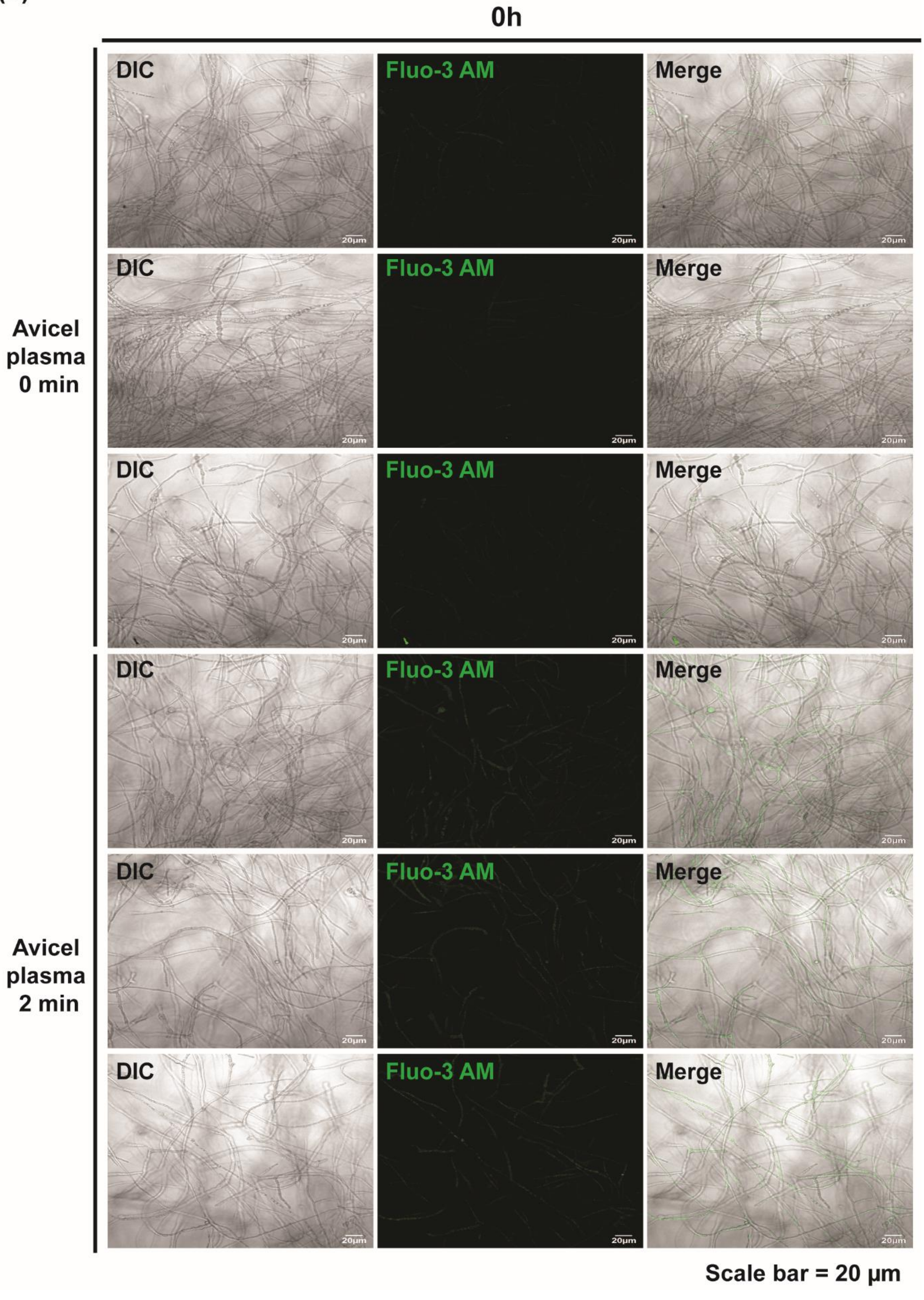
0h
Avicel plasma 0 min
Avicel plasma 2 min
DIC
Fluo-3 AM
Merge
20μm
Scale bar = 20 μm

(b)

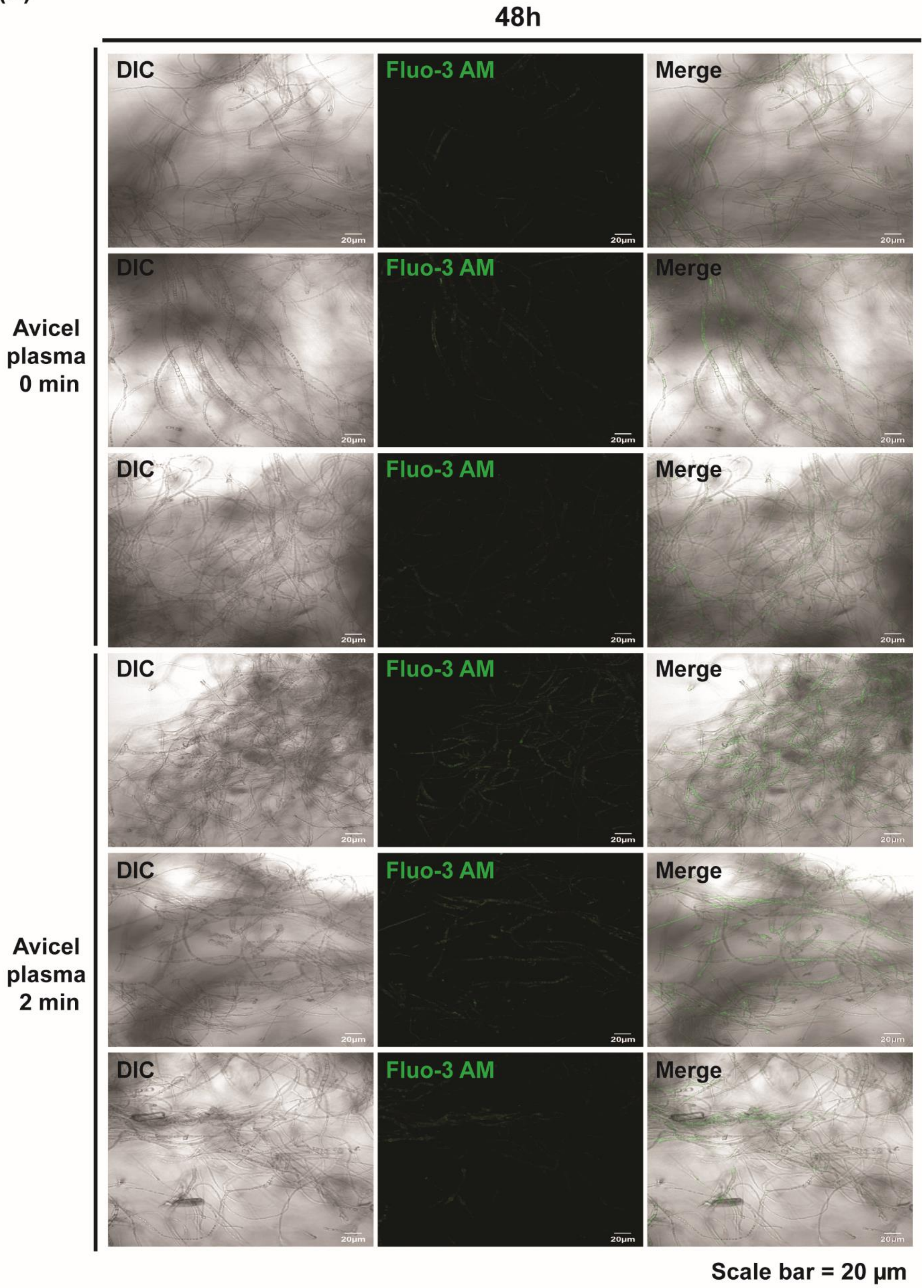
48h
DIC
Fluo-3 AM
Merge
Avicel plasma 0 min
Avicel plasma 2 min
20μm
Scale bar = 20 μm

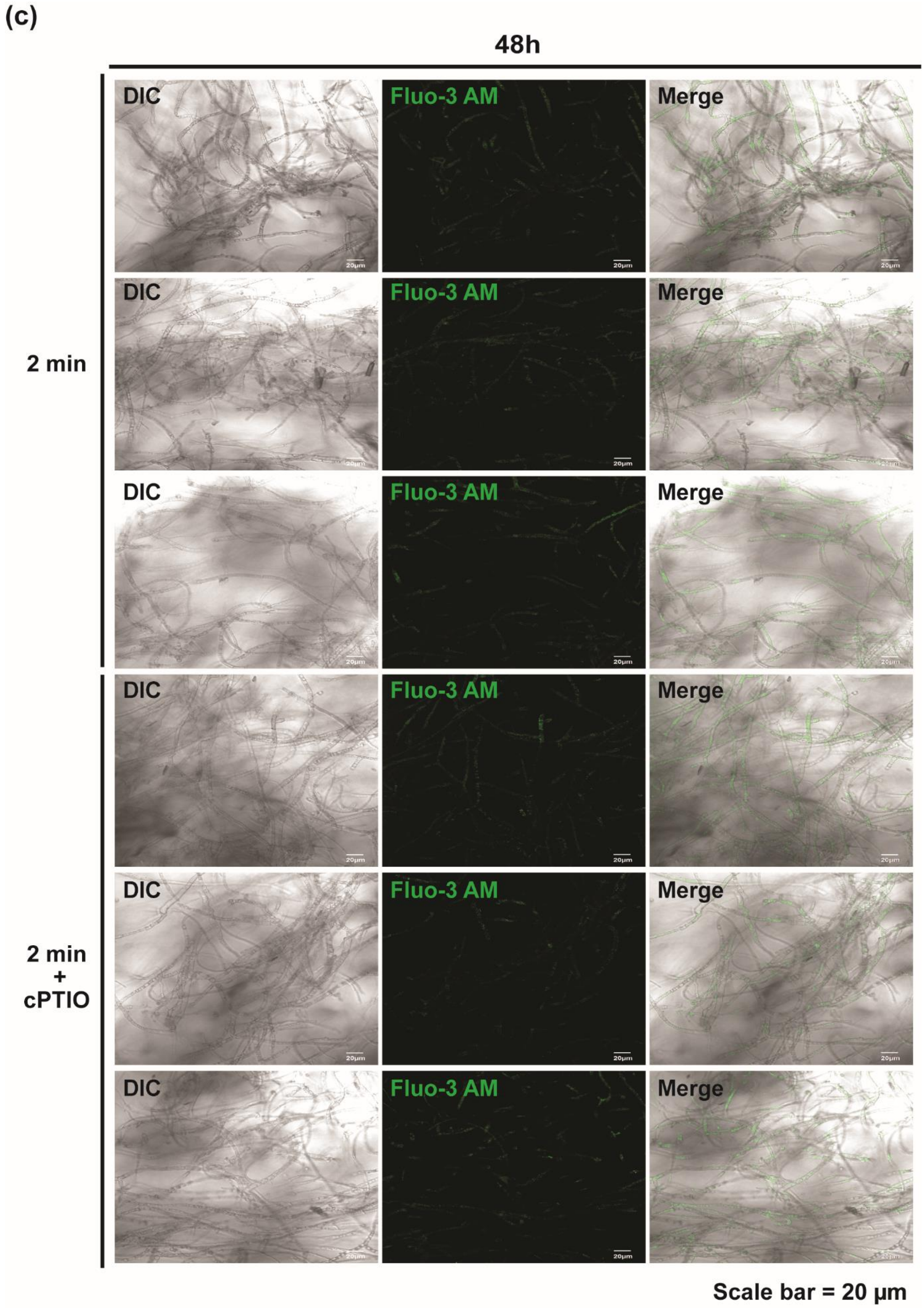
(c)
48h
DIC
Fluo-3 AM
Merge
2 min
2 min
+
cPTIO
Scale bar = 20 µm

Supplementary Figure S7. Intracellular Ca^{2+} level in *N. crassa* hyphae after plasma treatment for 0 min (control) or 2 min. Hyphae were stained with Fluo-3 AM (green fluorescence) after 0h (a) and 48h (b) of incubation in avicel media. (c) Hyphae stained with Fluo-3 AM after plasma treatment followed by incubation in avicel media containing 10mM cPTIO for 48 h. Pictures of fungal hyphae in 3 different areas were taken. DIC; Differential Interference Contrast, Fluo-3 AM; fluorescence, Merge; combined image of DIC and fluorescence.

(a)

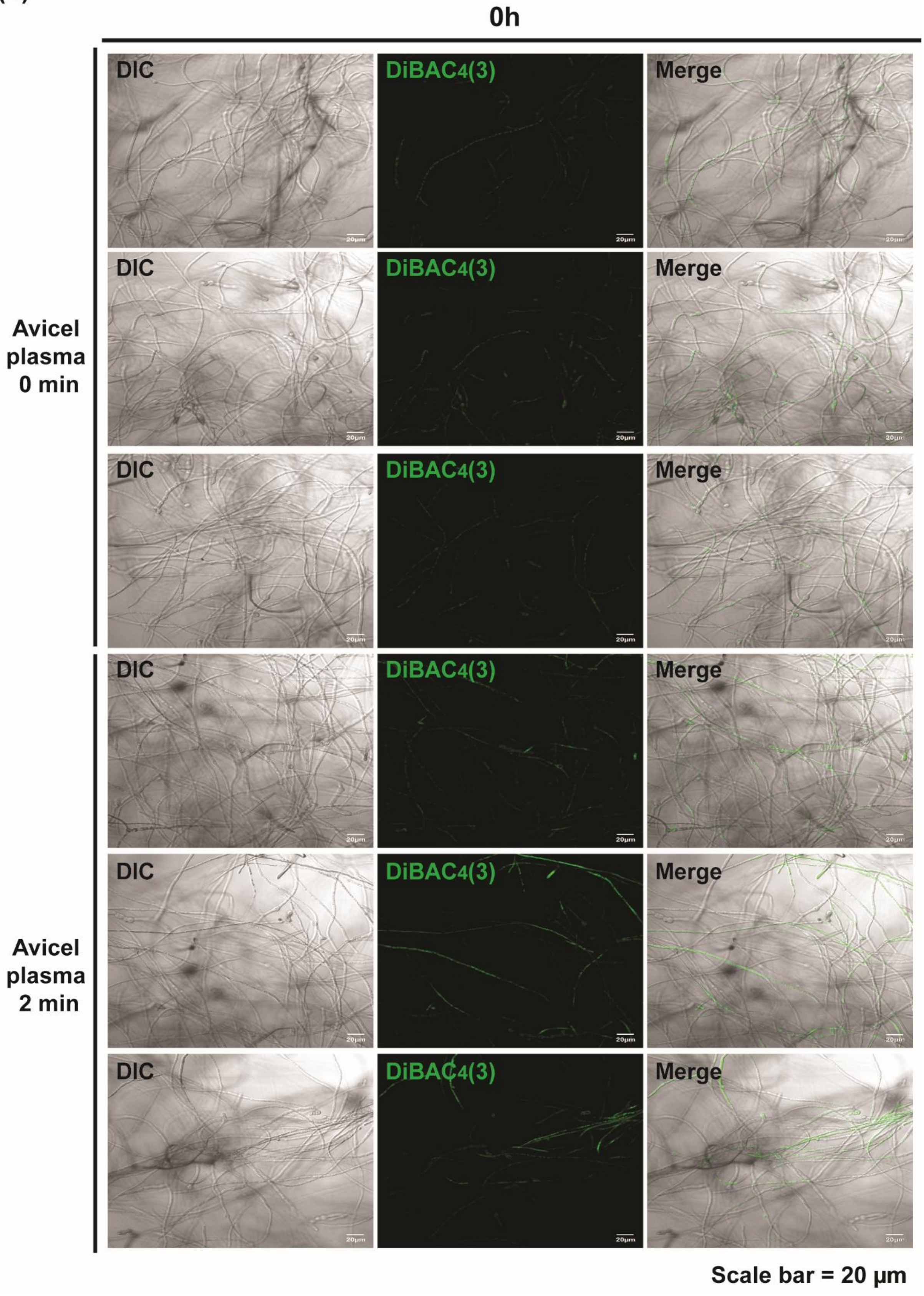
0h
Avicel plasma 0 min
Avicel plasma 2 min
DIC
DiBAC4(3)
Merge
20μm
Scale bar = 20 μm

(b)

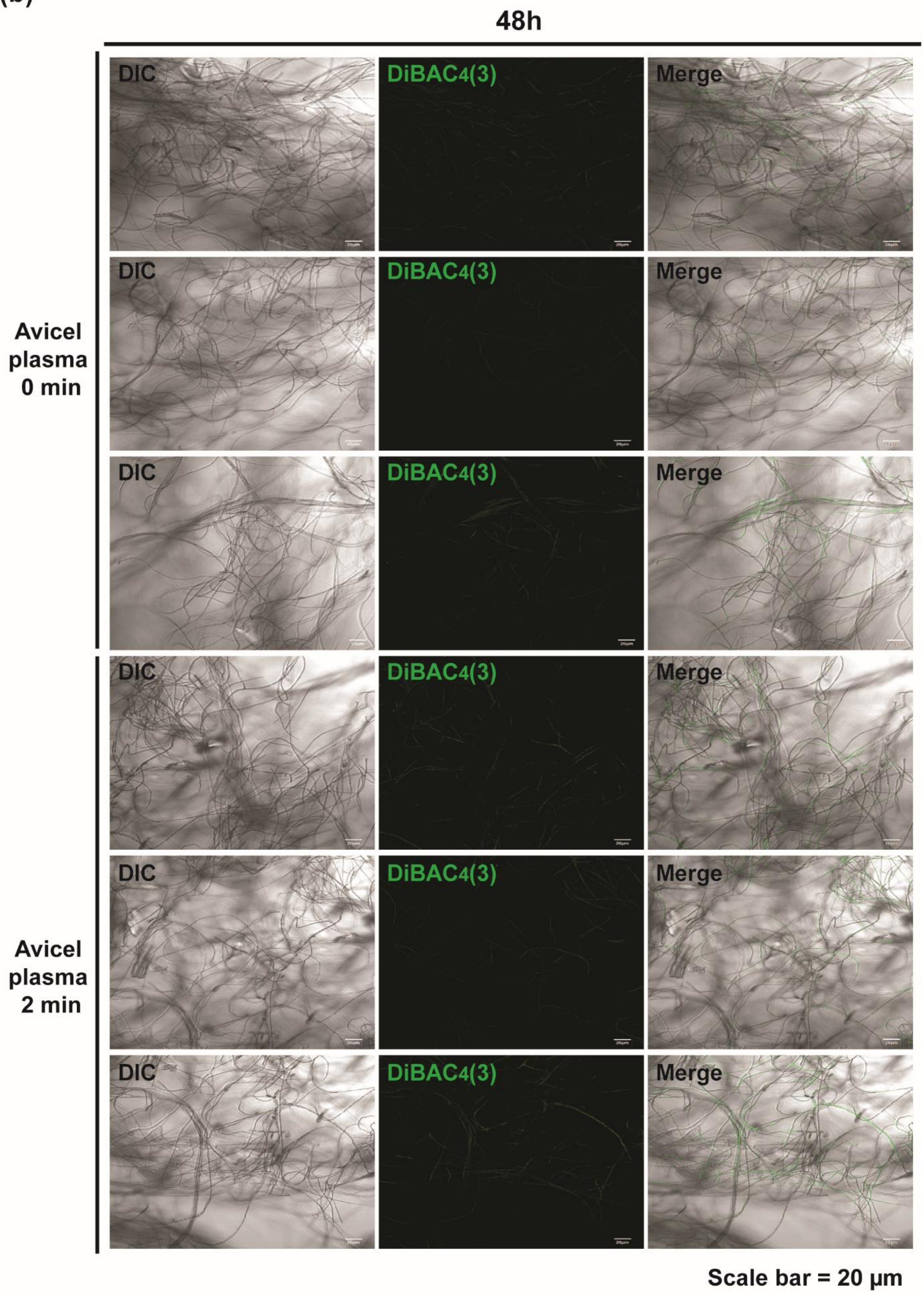
48h
DIC
DiBAC4(3)
Merge
Avicel plasma 0 min
Avicel plasma 2 min
Scale bar = 20 μm

Supplementary Figure S8. **Analysis of membrane potential in *N. crassa* hyphae after plasma treatment for 0 min (control) or 2min.** Hyphae were stained with DiBAC$_4$(3) (green fluorescence) after 0h (a) and 48h (b) of incubation in avicel media. Pictures of fungal hyphae in 3 different areas were shown. DIC; Differential Interference Contrast, DiBAC$_4$(3); fluorescence, Merge; combined image of DIC and fluorescence.
